# Transcriptomic analysis of early fruit development in Chinese white pear (*Pyrus bretschneideri Rehd.*) and functional identification of *PbCCR1* in lignin biosynthesis

**DOI:** 10.1186/s12870-019-2046-x

**Published:** 2019-10-11

**Authors:** Xueqiang Su, Yu Zhao, Han Wang, Guohui Li, Xi Cheng, Qing Jin, Yongping Cai

**Affiliations:** School of Life Science, Anhui Agricultural University, Hefei, Anhui China

**Keywords:** *Pyrus bretschneideri*, Stone cells, Lignin biosynthesis, Transcriptomic analysis, Cinnamoyl-CoA reductase

## Abstract

**Background:**

The content of stone cells and lignin is one of the key factors affecting the quality of pear fruit. In a previous study, we determined the developmental regularity of stone cells and lignin in ‘Dangshan Su’ pear fruit 15-145 days after pollination (DAP). However, the development of fruit stone cells and lignin before 15 DAP has not been heavily researched.

**Results:**

In this study, we found that primordial stone cells began to appear at 7 DAP and that the fruit had formed a large number of stone cells at 15 DAP. Subsequently, transcriptome sequencing was performed on fruits at 0, 7, and 15 DAP and identified 3834 (0 vs. 7 DAP), 4049 (7 vs. 15 DAP) and 5763 (0 vs. 15 DAP) DEGs. During the 7-15 DAP period, a large number of key enzyme genes essential for lignin biosynthesis are gradually up-regulated, and their expression pattern is consistent with the accumulation of lignin in this period. Further analysis found that the biosynthesis of S-type lignin in ‘Dangshan Su’ pear does not depend on the catalytic activity of *PbSAD* but is primarily generated by the catalytic activity of caffeoyl-CoA through CCoAOMT, CCR, F5H, and CAD. We cloned *PbCCR1*, *2* and analysed their functions in Chinese white pear lignin biosynthesis. *PbCCR1* and *2* have a degree of functional redundancy; both demonstrate the ability to participate in lignin biosynthesis. However, *PbCCR1* may be the major gene for lignin biosynthesis, while *PbCCR2* has little effect on lignin biosynthesis.

**Conclusions:**

Our results revealed that ‘Dangshan Su’ pear began to form a large number of stone cells and produce lignin after 7 DAP and mainly accumulated materials from 0 to 7 DAP. *PbCCR1* is mainly involved in the biosynthesis of lignin in ‘Dangshan Su’ pear and plays a positive role in lignin biosynthesis.

## Background

Pear belongs to the family Rosaceae and is an important fruit crop. Because of its rich nutrition and good taste, pear fruit is popular with the public, and pear tree has a notably wide planting area around the world [1]. Pear has more than 2000 years of cultivation history in China [2]. ‘Dangshan Su’ pear (*Pyrus bretschneideri* cv. Dangshan Su) is a high-quality cultivar native to Daishan County (Dangshan County, Anhui Province, China). It has a long history of cultivation and is known for its unique flavour, rich nutrition and high medicinal value [3–5]. The ‘Dangshan Su’ pear has excellent fruit quality, high yield and storage resistance, and it is a late-maturing variety. However, ‘Dangshan Su’ pear has the drawback of having a relatively high number of stone cells, which strongly affect the quality of the fruit.

Stone cells are unique to pear fruit, and they influence the unique flavour and quality of pear fruits. Excessive stone cell content in pear fruit not only affects the accumulation of sugar and other nutrients but also impairs taste. Stone cells are thick-walled tissue cells composed mainly of lignin and cellulose, and those of pear are classified as short stone cells [6]. Numerous studies have shown that stone cells are derived from primordial cells. At 15 DAP, the cell wall of the parenchyma cells was unevenly thickened, while a large amount of lignin was transported to the thickened secondary wall and deposited to form stone cells [7]. After the stone cells are formed, they aggregate into a cluster to form a stone cell mass. The peak period of stone cell formation is 15-67 DAP, and then, their content gradually decreases [6, 8]. In the development of pear stone cells, the thickening of secondary walls and the accumulation of lignin are two critically important steps [9]. In pear stone cells, lignin content accounts for approximately 20-30% of the total dry weight [10, 11]. Therefore, a systematic study of the thickening of the secondary walls of parenchyma cells and the biosynthesis of lignin in pear fruit have important implications for the molecular mechanisms of pear stone cell formation.

Lignin is the most important component of stone cells, accounting for 18% of the stone cell content [12]. Lignin monomers can be divided into three different types: G-units (guaiacyl lignin), S-units (syringyl lignin) and H-units (hydroxyphenyl lignin). G-unit lignin is synthesized from coniferyl alcohol, S-unit lignin is polymerized from sinapyl alcohol, and H-unit lignin is synthesized from *p*-coumaryl alcohol [13]. Previous studies have found that the fruit lignin of ‘Dangshan Su’ pear consists of two components, G and S [14]. Research on lignin biosynthesis has been underway for more than a century, and the known lignin biosynthetic pathway has been continuously improved [15–17]. First, L-phenylalanine (L-Phe) enters the lignin biosynthetic pathway. Phenylalanine ammonia-lyase (PAL) catalyses the formation of phenylacrylic acid from L-Phe (non-oxidative deamination) [18]. P-coumaric acid is synthesized from phenylacrylic acid in a process catalysed by cinnamate 4-hydroxylase (C4H) and 4-hydroxycinnamate-CoA ligase (4CL) [19, 20]. Then, caffeic acid 3-O-methyltransferase (COMT), caffeoyl-CoA O-methyltransferase (CCoAOMT), cinnamoyl-CoA reductase (CCR), cinnamyl alcohol dehydrogenase (CAD) and other key enzymes are responsible for the formation of lignin monomers [21–24]. The lignin monomers are gradually polymerized under the action of peroxidase (POD) and laccase (LAC) to form lignin complexes [25, 26]. The lignin produced by these complex catalytic reactions is deposited on the thicker secondary wall to form stone cells.

Transcriptome analysis can comprehensively examine the function and structure of genes and reveal the molecular mechanisms of specific biological processes [27]. Transcriptome analysis has been widely used in the study of various metabolic pathways in plants. For example, *Scutellaria viscidula* was subjected to transcriptome analysis to analyse the biosynthetic mechanism of flavonoids [28], and the differential genetic pathways and pathways related to drought tolerance in *Pennisetum glaucum* were explored [29]. In pear, Zhang et al. revealed the molecular mechanism of the difference in stone cells between ‘Dangshan Su’ pear and ‘Lianglizaosu’ by comparative transcriptome analysis [5]. However, this previous study of lignin in pear fruit examined only the 23-145 DAP period, during which a large number of stone cells are produced. Therefore, we here attempt to advance the time point of study to find the critical period when stone cells first begin to form in large quantities. This initial development period will allow more effective exploration of the molecular mechanisms of pear stone cell formation and lignin biosynthesis.

In this study, we used fruits obtained at 0, 7, and 15 DAP as experimental materials to investigate the mechanism of stone cell formation in the early stage of pear fruit development. First, the stone cell and lignin contents of fruits in three periods were compared. Subsequently, transcriptome sequencing was performed to analyse differential gene expression in the fruits of these three periods. Finally, the functions of two key enzymes (*PbCCR1* and *2*) were characterized to clarify their roles in lignin biosynthesis. Our results reveal the molecular mechanism of stone cell formation and development in pear fruit, providing a theoretical basis for ‘Dangshan Su’ pear molecular genetic breeding and variety improvement.

## Results

### Analysis of stone cells and lignin content in fruits

**Fig. 1:**
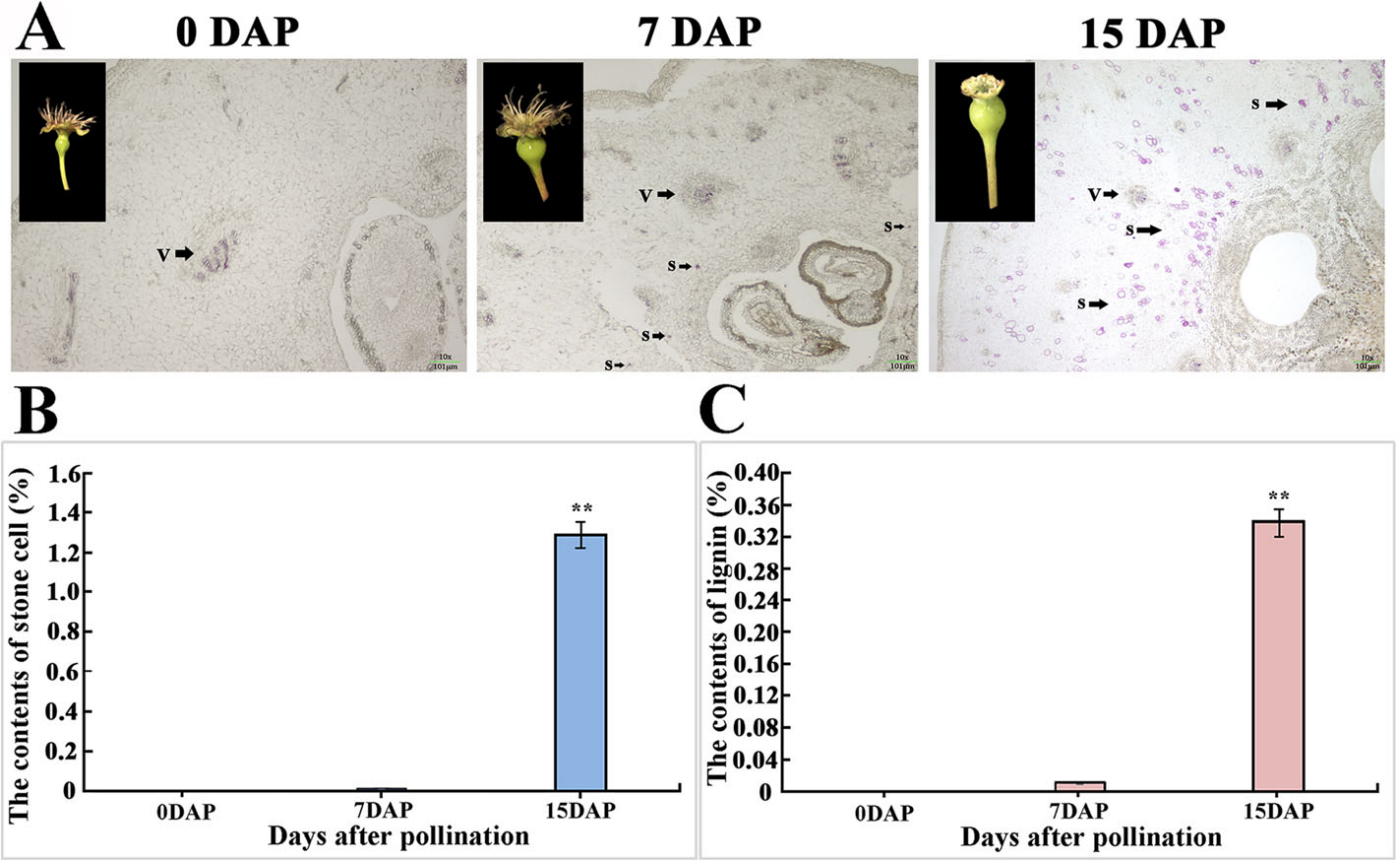
Investigation of stone cell and lignin contents in 0, 7, 15 DAP fruits. **a** Fruit morphology and stone cell phloroglucinol staining of 0, 7, and 15 DAP fruits were performed. **b** Stone cell contents of 0, 7, 15 DAP fruits. **c** Lignin contents of 0, 7, and 15 DAP fruits. **Significant differences between the stone cell or lignin levels of the different developmental periods (*P* < 0.01)

For the fresh samples obtained (0, 7, 15 DAP fruits), we first stained the samples with phloroglucinol and then performed sectioning. After staining with phloroglucinol, we found that the results of tissue sectioning showed significant differences. As shown in the Fig. 1a, the 0 DAP fruit was not dyed red by the phloroglucinol dye but was red only in the vascular bundle area. The staining results of the 7 DAP fruit were similar to those of the 0 DAP fruit, but we found stone cell primordia stained red near the core of the fruit. This finding indicates that a few stone cell primordia are present in this period, and the development of stone cells begins to enter a period of relatively rapid growth. In the 15 DAP fruit, we found a large number of stone cell clusters dyed red by phloroglucinol near the core and pulp, indicating that a large number of stone cells were formed between 7 and 15 DAP and aggregated to form stone cell clusters. Then, we measured the stone cell content and lignin content of the fruits in the three periods, and the measured results were consistent with the staining results (Fig. 1b, c). Stone cells were scarce in the 0 DAP samples, and notably few stone cells were identified in the 7 DAP samples, while the content of stone cells increased greatly in the 15 DAP samples. The change trend in lignin content was consistent with the change in stone cell content, which was also a gradually increasing process.

### Overview of RNA-Seq results

According to our research results, stone cells and lignin undergo considerable changes among the three time points 0, 7, 15 DAP, and the contents at 15 DAP exhibit a significant increase compared with the previous two periods. To clarify the transcriptional changes in ‘Dangshan Su’ fruit in the early stages of stone cell development, fruits from 0, 7, 15 DAP were selected for comparative analysis by Illumina sequencing, and three biological replicates were evaluated for each time point. In this study, we extracted total RNA from all nine samples (Additional file 9: Figure S1) and then constructed cDNA transcriptome sequencing library. Primary component analysis (PCA) and duplicate correlation checking scatter plot analysis showed high correlations among the three biologically replicated samples in each group (Additional file 10: Figure S2, Additional file 11: Figure S3). After trimming the raw data (deleting adaptors and low-quality sequences), 0 DAP fruit obtained the most reads (50313188, 53291526, 51885204), followed by 15 DAP fruit (45576288, 40812768, 45463438), 7 DAP fruit obtained the least reads (35573064, 31374310, 38135470) (Table 1). In addition, We found that the GC content of 9 libraries was in 48.27-49.11%, while the Q20 and Q30 values were greater than 96.37 and 91.75%, respectively. Because of the high quality of the sequencing results, most reads could be mapped to the reference genome of ‘Dangshan Su’. The percentages of mapped reads for the libraries were highly similar (about 80%), and the ratios of multiply mapped reads were 8.35-9.77%. The reads mapped to ‘+’ and reads mapped to ‘-’ showed greater than 35% coverage at all samples. The statistical results of the genome structure distribution showed that each sample had a very high similarity (Additional file 12: Figure S4). In single-site mapping, the intergenic region was the largest component. Of the total mapped reads, 56.36-58.80% of reads were mapped to intergenic regions, and 40.13-42.60% of reads were mapped to exon regions. The intron region had the smallest percentage, with only approximately 0.99-1.06% of reads being mapped to this region. Taken together, these results indicated that the RNA-Seq sequencing results were reliable and could be further analysed.

**Table 1:** Summary of the RNA-Seq data in three stages of ‘Dangshan Su’ fruit

ID	‘Dangshan Su’ fruit of 0 DAP			‘Dangshan Su’ fruit of 7 DAP			‘Dangshan Su’ fruit of 15 DAP		
	0 DAP-1	0 DAP-2	0 DAP-3	7 DAP-1	7 DAP-2	7 DAP-3	15 DAP-1	15 DAP-2	15 DAP-3
Total reads	50313188	53291526	51885204	35573064	31374310	38135470	45576288	40812768	45463438
Total mapped	40506401 (80.51%)	42693772 (80.11%)	41596368 (80.17%)	28379560 (79.78%)	25200858 (80.32%)	30434463 (79.81%)	36456831 (79.99%)	32775750 (80.31%)	36565932 (80.43%)
GC Content	48.27%	48.92%	49.11%	48.61%	48.42%	48.56%	48.78%	48.59%	48.40%
Q20a	98.45%	98.54%	98.43%	96.60%	96.74%	96.63%	96.64%	96.37%	96.49%
Q30b	94.63%	94.82%	94.65%	92.44%	92.60%	92.17%	92.13%	91.75%	92.05%
Mutiple mapped	9.76%	9.77%	9.71%	8.70%	8.74%	8.62%	8.42%	8.38%	8.35%
Uniquely mapped	70.75%	70.35%	70.46%	71.08%	71.25%	71.19%	71.90%	71.93%	72.08%
Reads map to ‘+’	35.49%	35.32%	35.37%	35.62%	35.71%	35.66%	35.97%	35.99%	36.06%
Reads map to ‘-’	35.26%	35.03%	35.09%	35.46%	35.55%	35.53%	35.92%	35.94%	36.02%
Non-splice reads	45.70%	44.71%	44.77%	45.50%	45.57%	45.44%	46.13%	46.15%	45.99%
Splice reads	25.05%	25.63%	25.69%	25.58%	25.69%	25.75%	25.77%	25.78%	26.09%

### Gene expression analysis and cluster analysis of DEGs

According to the relationship between gene transcripts per million (TPM) deviation within 15% of the final value (based on 100% mapped reads) and percentage of mapped reads, when all samples were 3.5 < TPM < 15, the curve reached saturation state (Additional file 13: Figure S5). This result showed that our sequencing depth was good and could be used for gene expression analysis. By analysing the density distributions of gene expression, we gained a macroscopic understanding of the gene expression levels in these samples (Additional file 14: Figure S6). In the 0 DAP samples, the number of genes with low expression levels (log2(TPM) < − 1) was higher than those in the other two samples (7 DAP and 15 DAP), but the number of genes with high expression levels (log2(TPM) > 1) was significantly lower than those of the other two samples. However, the number of highly expressed genes at 7 DAP was less than that at 15 DAP. Therefore, low-expression genes were the most abundant at 0 DAP, while high-expression genes were significantly enriched at 15 DAP. Similar results were obtained in the hierarchical clustering heat map of transcript expression (Additional file 15: Figure S7). In the heat map, we can clearly observe that the transcripts of the three parallel samples in each group showed largely the same expression levels. However, the transcript expression levels changed with fruit development. Among the 0 DAP samples, a large number of transcripts (probably 60-70%) showed low expression levels, which represented the greatest proportion among the three groups. At 7 DAP compared with 0 DAP, the number of transcripts with low expression and high expression levels was not notably different, but more transcripts were observed at the medium expression level. Clearly, 15 DAP was the group with the highest expression of transcripts, and approximately 60% of its transcripts exhibited high expression levels.

**Fig. 2:**
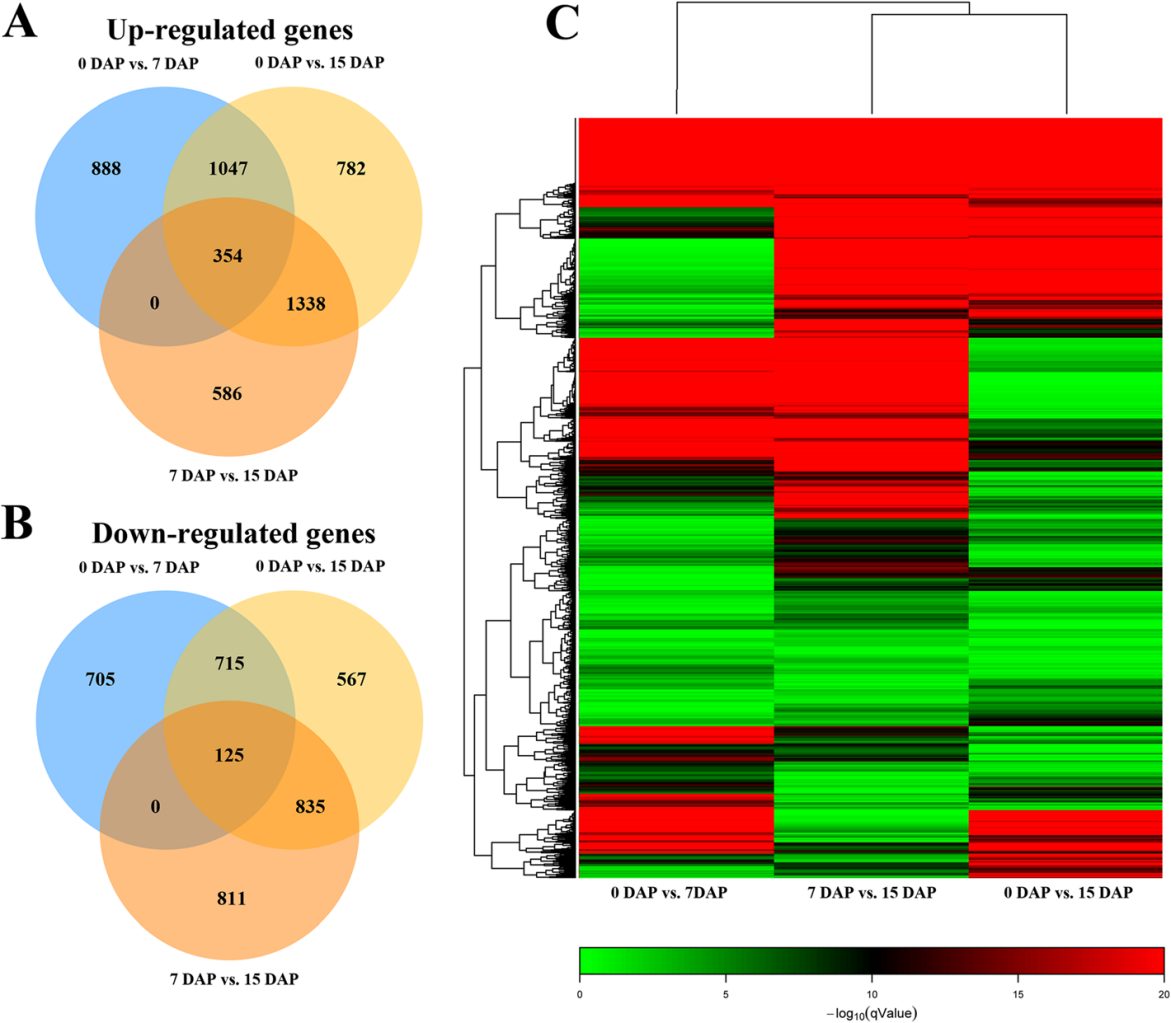
Venn diagram of significantly up-regulated and down-regulated genes in 0, 7, 15 DAP fruits and cluster analysis of DEG expression based on log ratio q-value data. **a**, **b** Venn diagram analysis of stone cell development-related DEGs. **c** Clustering analysis of DEGs based on fold change

After analysing the expression patterns of each group of transcripts, we summarized the numbers of DEGs in the three stages of fruit development. In all pairwise comparisons, a total of 5763 DEGs were identified in 0 DAP vs. 15 DAP, making this the most abundant group (3521 up-regulated and 2242 down-regulated). We identified 3834 DEGs in the 0 DAP vs. 7 DAP group (2289 up-regulated and 1545 down-regulated). A total of 4049 DEGs (2278 up-regulated and 1771 down-regulated) were discovered in the 7 DAP vs. 15 DAP group (Additional file 16: Figure S8). Venn diagrams were used to show the DEGs among all pairwise comparisons (Fig. 2a, b). In all pairwise comparisons, a total of 354 DEGs were up-regulated, and 125 DEGs were down-regulated. However, we found some genes that were specifically differentially expressed. For example, we identified 1593 distinctive DEGs in the group between the 0 DAP and 7 DAP stages (888 up-regulated and 705 down-regulated), while we found only 1397 (586 up-regulated and 811 down-regulated) and 1349 (782 up-regulated and 567 down-regulated) distinctive DEGs in the 7 DAP vs. 15 DAP and 0 DAP vs. 15 DAP groups, respectively. The greatest number of down-regulated genes was identified in the 0 DAP vs. 15 DAP group (2242), and the proportion of strongly down-regulated DEGs (with high expression fold changes) was also the highest among all the pairwise comparisons (Fig. 2c). Interestingly, in this group, we found that although it contained the greatest number of up-regulated genes, the proportion of strongly up-regulated DEGs was not the highest. Instead, the proportion of strongly up-regulated DEGs was the highest of the three groups in the 7 DAP vs. 15 DAP group.

### Functional annotation and pathway enrichment analysis of DEGs

To explore the molecular mechanism of stone cell development in ‘Dangshan Su’ pear, we used the EuKaryotic Orthologous Groups (KOG), Gene Ontology (GO) and Kyoto Encyclopedia of Genes and Genomes (KEGG) databases to analyse DEGs. The results of the KOG enrichment analysis showed that the DEGs were mainly annotated in general function prediction only (R), posttranslational modification (O), signal transduction mechanisms (T), carbohydrate transport and metabolism (G), and amino acid transport and metabolism (E). Twenty-five KOG functional categories were represented (Additional file 17: Figure S9). However, we did not find any DEGs that were annotated in the cell motility (N) classes. In the three groups of pairwise comparisons, 157 (8.4%), 173 (8.4), and 211 (7.2%) DEGs were annotated to involve the function of secondary metabolite biosynthesis (Q class).

To further understand the functions of the DEGs, we also performed GO classification analysis (Additional file 18: Figure S10). Specifically, for 0 DAP vs. 7 DAP, 7 DAP vs. 15 DAP and 0 DAP vs. 15 DAP, the three groups had 3061 (79.8), 3290 (81.3%) and 4667 (80.1%) DEGs, respectively, annotated in the GO database. These DEGs were enriched in the three major functional categories of biological process (BP), cellular component (CC) and molecular function (MF). The above three functional categories could be divided into 67 more detailed subcategories, including 27 (BP1-27), 22 (CC1-22), and 18 (CC1-18) functional subcategories. Cellular process (BP-22), cell (CC-16), and binding (MF-15) were the most important functional subcategories of the three major functional categories in addition to these large numbers of functional subcategories. In all pairwise comparisons, significantly higher amounts of the up-regulated DEGs were annotated in single-organism process (BP-1), metabolic process (BP-8), membrane (CC-13), organelle (CC-17), catalytic activity (MF-4), and nucleic acid binding transcription factor activity (MF-1); the down-regulated DEGs were annotated in biological regulation (BP-19), response to stimulus (BP-4), membrane part (CC-11), organelle part (CC-18), transporter activity (MF-3), and signal transducer activity (MF-10).

**Fig. 3:**
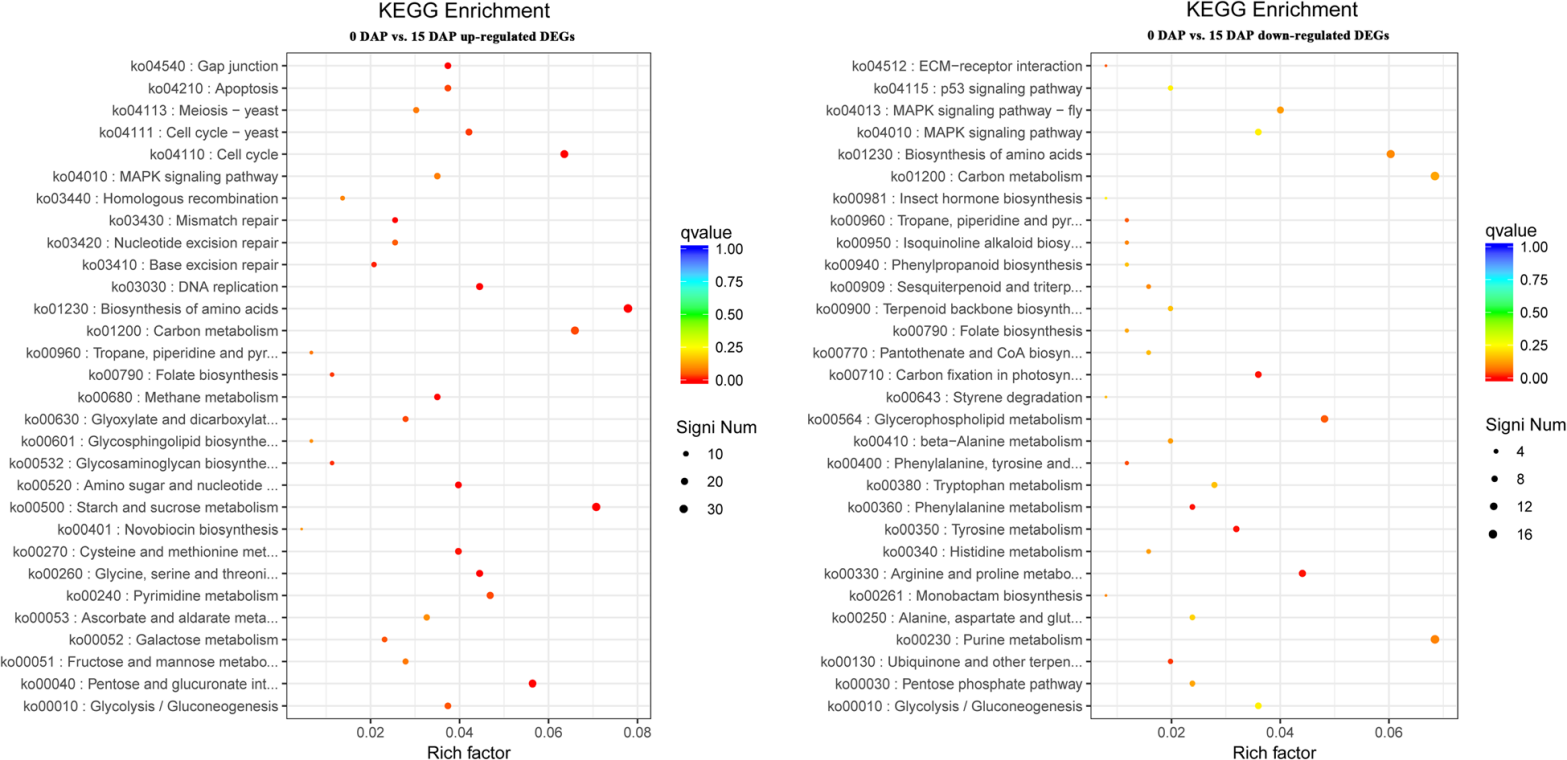
KEGG enriched scatter plots of up-regulated (left) and down-regulated (right) DEGs in the 0 DAP vs. 15 DAP comparison. Rich factor refers to the ratio of the number of DEGs enriched in the pathway to the number of annotated genes. The larger the Rich factor, the more significant the enrichment is. The q-value is the corrected *P*-value after multiple hypothesis testing, which ranges from 0 to 1. The closer to zero, the more significant the enrichment is

Mapping DEGs to the KEGG database allows for the identification of significant metabolic or signalling pathways in which DEGs are enriched. The 0 DAP vs. 15 DAP group had 660 DEGs annotated to 177 KEGG pathways (Additional file 19: Figure S11, Additional file 2: Table S2). These KEGG pathways involved four major functional groups: cellular processes, environmental information processing, genetic information processing, and metabolism, and these functional groups were divided into four, three, four, and twelve functional subgroups, respectively (Additional file 3: Table S3). In the 0 DAP vs. 15 DAP group, the up-regulated DEGs were mainly concentrated in pathways such as carbon metabolism (ko01200), pyrimidine metabolism (ko00240), and biosynthesis of amino acids (ko01230); the down-regulated DEGs were concentrated in purine metabolism (ko00230), retinol metabolism (ko00830), glycerophospholipid metabolism (ko00564) and other KEGG pathways (Fig. 3). In addition, we found that some DEGs were enriched in the pathways of apoptosis (ko04210), phenylalanine metabolism (ko00360, ko00400) and phenylpropanoid biosynthesis (ko00940) (Fig. 3, Additional file 2: Table S2).

### DEGs associated with stone cell lignin biosynthesis

The phenylpropanoid metabolism pathway is usually closely related to the biosynthesis of secondary walls and lignin [30]. RNA-seq data analysis showed that active transcription and expression were detected for some key enzyme genes in the phenylpropanoid metabolism pathway that control the entry of lignin biosynthesis carbon sources, including PAL (3), C4H (3), 4CL (2), HCT (5), CCoAOMT (1), F5H (2), CCR (3), CAD (3), POD (3) and other key enzyme genes in lignin biosynthesis (Additional file 20: Figure S12, Table 2). Coumarin and lignin are closely related in the phenylpropanoid metabolism pathway with a common precursor substance (coumaric acid). Interestingly, we did not identify the presence of DEGs in the coumarin biosynthetic pathway (Additional file 20: Figure S12).

**Table 2:** Expression level of key enzymes in lignin biosynthesis pathway of ‘Dangshan Su’ pear

Gene name	Genome ID	0 DPA VS 7 DPA TPM			7 DPA VS 15 DPA TPM			0 DPA VS 15 DPA TPM		
		0 DPA	7 DPA	DEGs	7 DPA	15 DPA	DEGs	0 DPA	15 DPA	DEGs
*PbPAL1*	Pbr008363.1	55.552	156.094	UP	156.094	275.613	UP	55.552	275.613	UP
*PbPAL2*	Pbr008387.1	0.0001	0.0001	*	0.0001	0.0001	*	0.0001	0.0001	*
*PbPAL3*	Pbr016460.1	27.557	53.181	*	53.181	108.033	UP	27.557	108.033	UP
*PbC4H1*	Pbr017290.1	300.966	259.520	*	259.520	1299.55	UP	300.966	1299.55	UP
*PbC4H2*	Pbr013141.1	406.084	370.695	*	370.695	360.987	*	406.084	360.987	*
*PbC4H3*	Pbr013138.1	35.905	37.884	*	37.884	5.084	DOWN	35.905	5.084	DOWN
*PbC3H*	Pbr020891.1	0.164	0.0001	*	0.0001	6.129	UP	0.164	6.129	UP
*Pb4CL1*	Pbr024635.1	3.706	8.496	UP	8.496	82.318	UP	3.706	82.318	UP
*Pb4CL2*	Pbr012851.1	34.962	158.292	UP	158.292	391.148	UP	34.962	391.148	UP
*PbCCoAOMT*	Pbr034039.1	6.174	19.613	*	19.613	389.731	UP	6.174	389.731	UP
*PbHCT2*	Pbr005360.1	17.081	8.925	*	8.925	34.744	UP	17.081	34.744	UP
*PbHCT17*	Pbr006709.1	1.926	109.163	UP	109.163	181.963	*	1.926	181.963	UP
*PbHCT18*	Pbr006711.1	0.0001	0.638	*	0.638	2.037	*	0.0001	2.037	*
*PbHCT49*	Pbr022422.1	808.729	1403.77	*	1403.77	574.853	DOWN	808.729	574.853	*
*PbHCT50*	Pbr022425.1	13.861	66.308	UP	66.308	210.283	UP	13.861	210.283	UP
*PbF5H1*	Pbr040547.1	0.381	0.842	*	0.842	31.635	UP	0.381	31.635	UP
*PbF5H2*	Pbr022142.1	1.748	4.611	*	4.611	103.477	UP	1.748	103.477	UP
*PbCCR1*	Pbr022402.1	14.366	34.882	UP	34.882	141.894	UP	14.366	141.894	UP
*PbCCR2*	Pbr022405.1	35.409	76.935	UP	76.935	165.976	UP	35.409	165.976	UP
*PbCCR3*	Pbr022403.1	0.157	0.338	*	0.338	0.719	*	0.157	0.719	*
*PbCOMT1*	Pbr034039.1	6.174	19.613	UP	19.613	389.731	UP	6.174	389.731	UP
*PbCOMT3*	Pbr038709.1	30.472	77.014	UP	77.014	483.801	UP	30.472	483.801	UP
*PbCAD1*	Pbr040236.1	0.237	0.108	*	0.108	0.0001	*	0.237	0.0001	*
*PbCAD2*	Pbr026287.1	7.771	10.568	*	10.568	29.151	UP	7.771	29.151	UP
*PbCAD3*	Pbr026289.1	2.318	12.256	UP	12.256	1.487	DOWN	2.318	1.487	*
*PbLAC1*	Pbr003857.1	0.097	3.876	*	3.876	197.086	UP	0.097	197.086	UP
*PbLAC14*	Pbr018935.1	0.051	0.018	*	0.018	1.384	*	0.051	1.384	*
*PbPOD1*	Pbr035186.1	485.635	299.328	*	299.328	398.483	*	485.635	398.483	*
*PbPOD2*	Pbr031894.1	270.852	1455.64	UP	1455.64	2402.79	*	270.852	2402.79	UP
*PbPOD3*	Pbr034480.1	0.643	3.399	*	3.399	3.586	*	0.643	3.586	*
*PbSAD*	Pbr004675.1	0.0001	0.399	*	0.399	2.777	*	0.0001	2.777	*
*PbUGT1*	Pbr005011.1	0.278	0.0001	*	0.0001	0.0001	*	0.278	0.0001	*
*PbUGT2*	Pbr005013.1	0.0001	0.0001	*	0.0001	0.0001	*	0.0001	0.0001	*
*PbUGT3*	Pbr005014.1	2.889	5.195	*	5.195	2.077	DOWN	2.889	2.077	*

*Indicates that the gene is not differentially expressed gene

Based on the results of transcriptome sequencing, we calculated the relative expression levels of these key lignin biosynthetic enzyme genes. In the 0 DAP vs. 7 DAP group, most of the key lignin biosynthetic enzyme genes had low expression levels. However, some key enzymes also showed up-regulated expression trends, such as *PbPAL1*, *Pb4CL1*, *2*, *PbCCR1*, *2*, *PbCOMT1*, *3*, *PbCAD3* and *PbPOD2*. It is worth mentioning that *PbPOD2* is the key enzyme gene in this stage that is related to the polymerization of lignin monomers, and it showed up-regulated expression (fragments per kilobase of exon per million mapped reads (TPM) value as high as 1455.64 at 7 DAP). At the 7-15 DAP stage, at least one member of each key lignin biosynthetic enzyme gene showed an upregulation trend (Table 2).

**Fig. 4:**
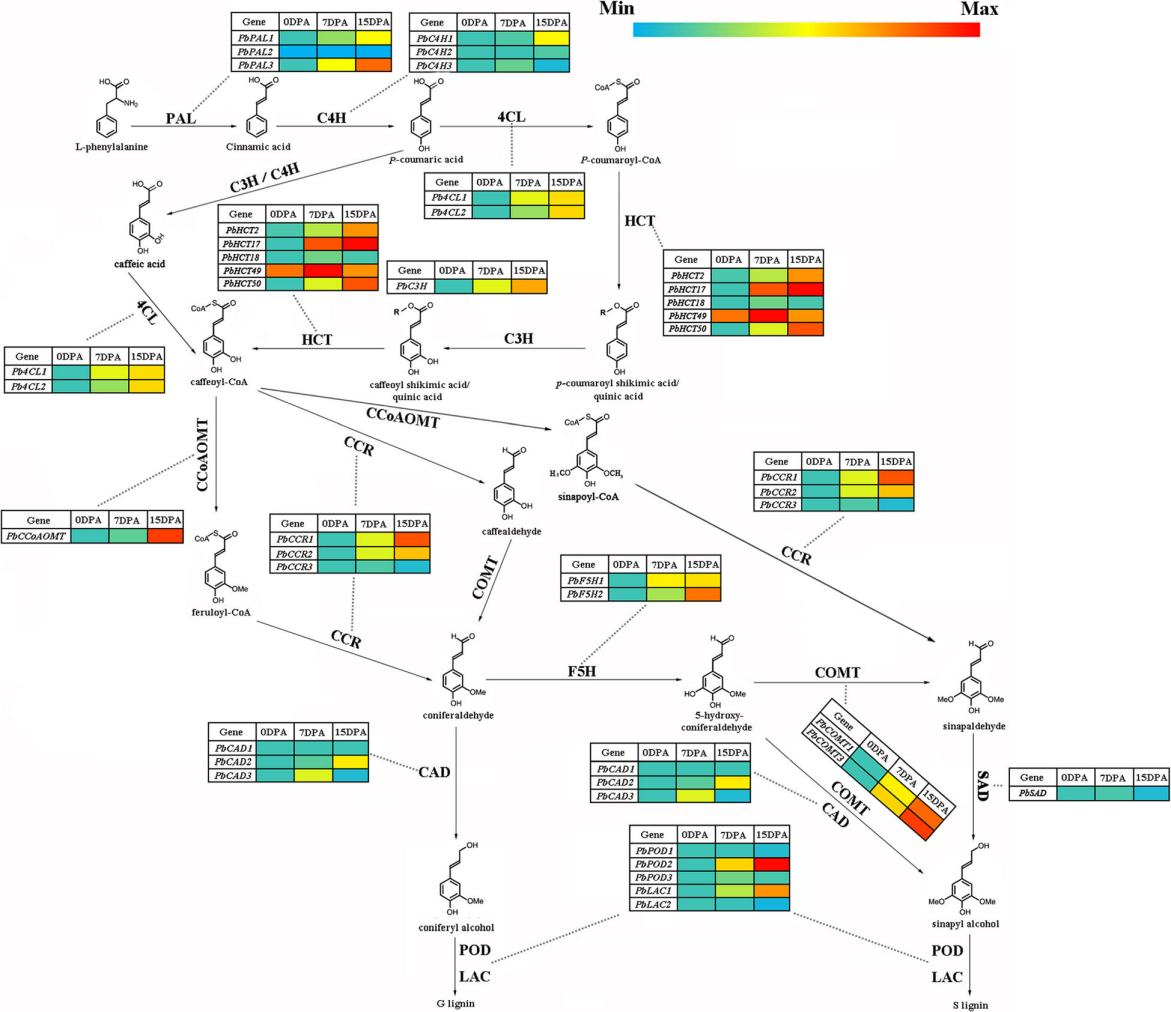
Expression patterns of key enzymes in lignin biosynthesis by qRT-PCR

We then performed qRT-PCR analysis on these key enzyme genes. We found that the results were consistent with the RNA-seq sequencing results and mapped the results to the lignin biosynthetic metabolic pathway (Fig. 4). L-Phe, as a precursor of lignin biosynthesis, flows into the metabolic pathway. The expression of *PbPAL1* was up-regulated at first, and *PbPAL2* rapidly increased in expression level after 7 DAP. These genes, including *PbC4H1*, *2*, *PbC3H*, *PbCCoAOMT*, *PbF5H1*, *2* and *PbCAD2*, exhibited similar expression patterns. These genes were expressed at lower levels before 7 DAP, but their expression after 7 DAP significantly and continuously increased, reaching the range of up-regulated differential expression. In addition, some genes began to increase from 0 DAP, such as *Pb4CL1*, *2*, *PbCCR1*, *2*, and *PbCOMT1*, *3*. In contrast, genes such as *PbCCR3* and *PbCAD1* showed extremely low expression levels during the whole period from 0 to 15 DAP. Finally, of the genes responsible for the polymerization of lignin monomers, only *PbLAC1* and *PbPOD2* showed upregulation of transcript levels. Overall, the expression levels of most key enzyme genes in lignin biosynthesis at the 0-7 DAP stage were low, and their expression levels continued to increase significantly from 7 DAP.

To further explore candidate genes related to stone cell development in pear fruits, 354 up-regulated DEGs were identified as common among the three groups. These DEGs include Dirigent-like protein (DIR, Pbr000712.1), KIP1-like protein (KIP1, Pbr001414.1), S-adenosylmethionine synthetase (S-AdoMet, Pbr001686.1), Glutamine amidotransferase (GATase, Pbr002524.1), GMC oxidoreductase (GMC oxred, Pbr011059.2), EamA-like transporter protein (EamA, Pbr024589.1), Terpene synthase (TPS, Pbr024829.1), Multicopper oxidase (Cu-oxidase, Pbr025074.1) and other genes. Both the transcriptome sequencing data and qRT-PCR results showed that these genes were up-regulated from 0 to 15 DAP (Additional file 21: Figure S13, Additional file 4: Table S4). This finding suggests that these genes may play an important role in the development and biosynthesis of ‘Dangshan Su’ pear stone cells and lignin.

Numerous studies have shown that transcription factors regulate the formation of plant cell walls and the biosynthesis of lignin [31–33]. Thus, we explored the expression patterns of three transcription factor families (MYB, AP2/ERF and NAC) (Additional file 22: Figure S14). In the MYB transcription factor family [34], we found 16 MYB gene family members that were gradually up-regulated and 8 that were gradually down-regulated. In addition, four MYB genes (*PbMYB47*, *73*, *93*, *125*) showed a pattern of expression that decreased first and then increased. Fourteen members of the AP2/ERF gene family [35] showed up-regulated expression, whereas only four genes showed a progressively down-regulated expression pattern. The expression of the NAC gene family [36] seemed to be relatively silent from 0 to 15 DAP, while the expression of only 7 family members was gradually up-regulated.

### Cinnamoyl-CoA reductase involved in lignin biosynthesis

**Fig. 5:**
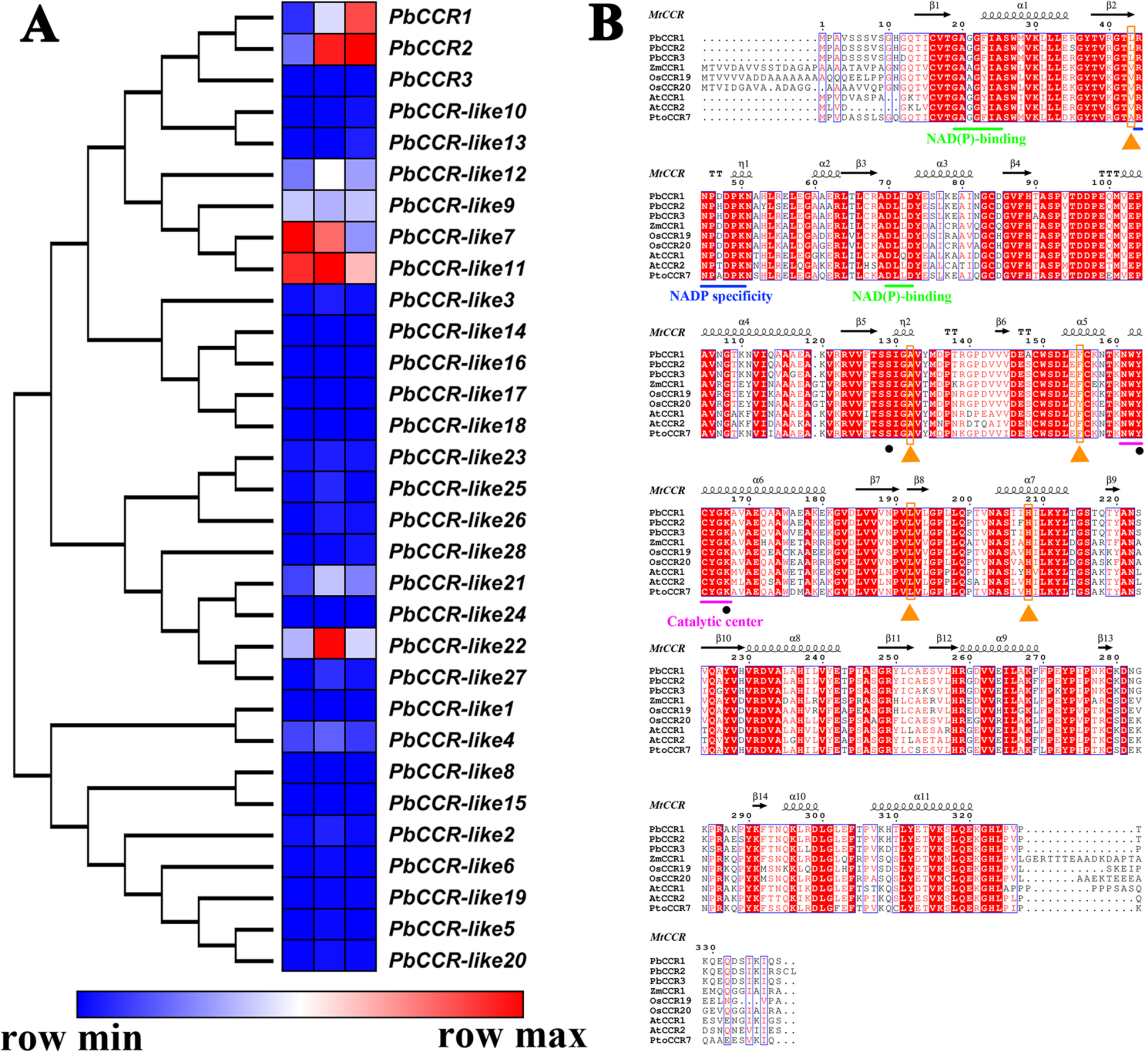
Expression profiles of 31 CCR genes in ‘Dangshan Su’ pear and sequence alignment of *PbCCR1*, *2*, *3* against genes from other plant species. **a** Analysis of the expression patterns of 31 CCR genes. **b** Using the crystal structure of *MtCCR* as a template, the blue underline indicates the NADP specificity site, and the green underline indicates the NAD(P) binding site. Black circles indicate catalytic triads. Orange triangles represent key catalytically active amino acid residues. The purple underline indicates the catalytic centre. The black wavy lines and arrows represent α-helices and β-sheets, respectively. *ZmCCR1* (CAA74071); *OsCCR19* and *20* (Os09g25150, Os08g34280); *AtCCR1* and *2* (AAG46037, AAG53687); *PtoCCR7* (KF145198)

The catalytic formation of cinnamoyl-CoA into cinnamaldehyde is a key step before the formation of lignin monomers. The CCR gene plays a major role in this critical step [37]. According to previous research results, 31 CCR genes were retained in the genome of Chinese white pear, and these CCR genes have conserved domains in this family [24]. We validated the accuracy of our transcriptome sequencing data by qRT-PCR experiments. After that step, we collected the FPKM values of all 31 CCR genes at 0, 7, 15 DAP and used the data to analyse the expression profiles of these genes (Fig. 5a). Approximately 74.2% of the CCR genes showed very low transcript levels at the 0-15 DAP stage. The expression patterns of *PbCCR-like7*, *11* were similar; both gene expression levels were high at 0 DAP and then decreased gradually. The expression levels of *PbCCR-like12*, *22* increased first and then decreased, although they differed in that *PbCCR-like22* was highly expressed at 7 DAP. In contrast, the expression of *PbCCR-like9* decreased first and then increased, reaching a low level of expression at 7 DAP. According to our research results, the lignin content of pear fruit increased gradually during the 0-15 DAP period. Previous studies have speculated that *PbCCR1*, *2*, *3* may be related to lignin biosynthesis in pear fruit. However, the expression profiles and qRT-PCR results showed that only *PbCCR1*, *2* gradually increased in the 0-15 DAP period, while the expression of *PbCCR3* was not detected at this stage.

To further understand the characteristics of *PbCCR1*, *2*, *3*, we collected and aligned some CCR genes that have been proven to be related to lignin biosynthesis (Fig. 5b). The obtained protein sequence contains conserved G-X-X-G-X-X-A and D-X-X-D NAD(P) binding sites. At the same time, the presence of the R-X-X-X-X-X-K NADP specificity motif was identified; this particular sequence distinguishes the CCR gene from other NAD(H)-dependent short-chain dehydrogenase superfamily (SDR) genes. More importantly, the NWYCYGK motif was also identified. This motif is a CCR signature motif that is highly conserved among these protein sequences. The Ser-Tyr-Lys catalytic triad, which represents active residues in SDRs, was found and marked with black circles. In addition, yellow triangles were used to identify key amino acid residues closely related to catalytic reactions. These key catalytic sites are also conserved, but there are exceptions. For example, the amino acid residues at position 43 associated with substrate and cofactor binding showed lower conservation, but the three genes in Chinese white pears showed strong species specificity (all are Leu at this location). Additionally, at the substrate binding site, the amino acid residue at position 155 was highly conserved, and only in *OsCCR20* was the tyrosine (Y) at this site replaced by phenylalanine (F) [38].

### Heterologous overexpression of *PbCCR1* and *2* in Arabidopsis

**Fig. 6:**
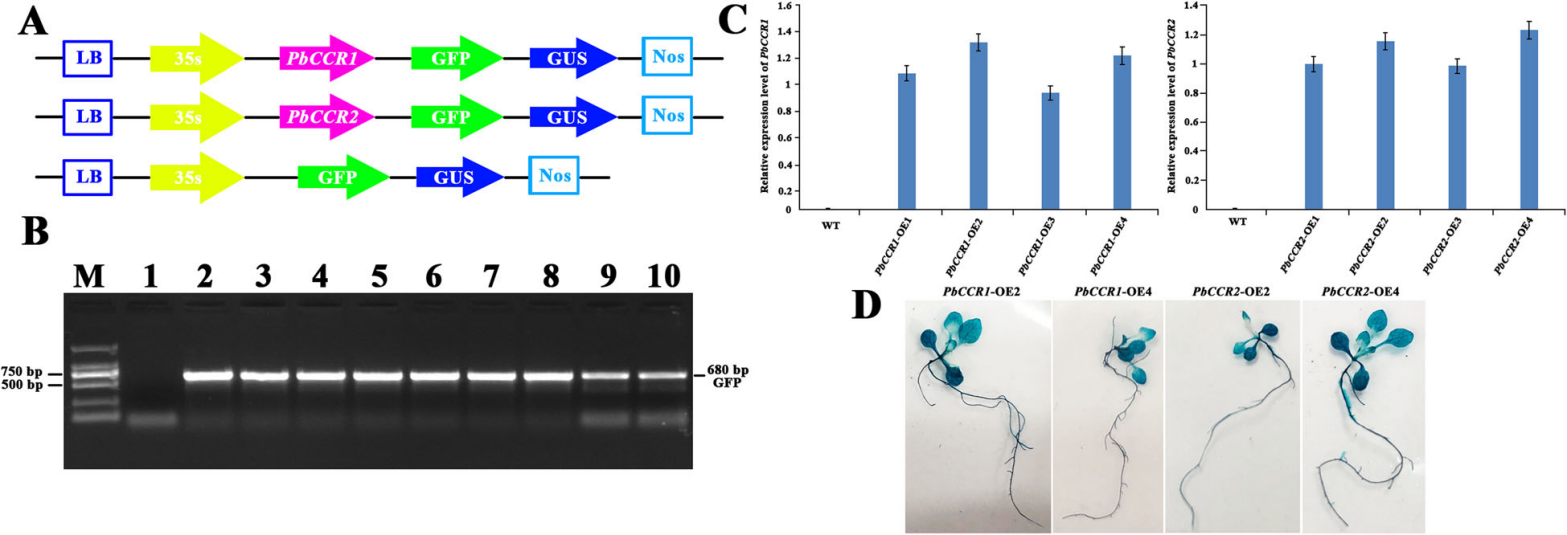
Overexpression of *PbCCR1* and *PbCCR2* in Arabidopsis. **a** Construction strategy of the plant expression vectors pCAMBIA1304-*PbCCR1* and *2*. **b** RT-PCR analysis for detecting the GFP gene in transgenic Arabidopsis. M: DL2000 DNA Marker; 1: negative control; 2: positive control; 3-6: *PbCCR1*-OE1-4 transgenic lines; 7-10: *PbCCR2*-OE1-4 transgenic lines. **c** The expression levels of *PbCCR1* and *2* in transgenic lines. Error bars represent the SD of three biological replicates. **d** β-Glucuronidase (GUS) histochemical staining of *PbCCR1*-OE2 and 4 and *PbCCR2*-OE2 and 4

We attempted to further explore the role of *PbCCR1* and *2* in lignin biosynthesis in pear fruit. First, we obtained the full-length sequences of these two genes. We ligated these two genes into the pCAMBIA1304 vector and obtained complete plant expression vectors, and we subsequently transformed wild-type Arabidopsis (Fig. 6a). Using RT-PCR for detection (Fig. 6b), we finally obtained 4 (*PbCCR1*-OE1-4) and 4 (*PbCCR2*-OE1-4) active transgenic lines. We detected the expression levels of *PbCCR1* and *2* in T3 generation transgenic plants. Based on the transcript levels (Fig. 6c, d), we found that the *gusA* gene had higher expression in *PbCCR1*-OE2, 4 and *PbCCR2*-OE2, 4.

**Fig. 7:**
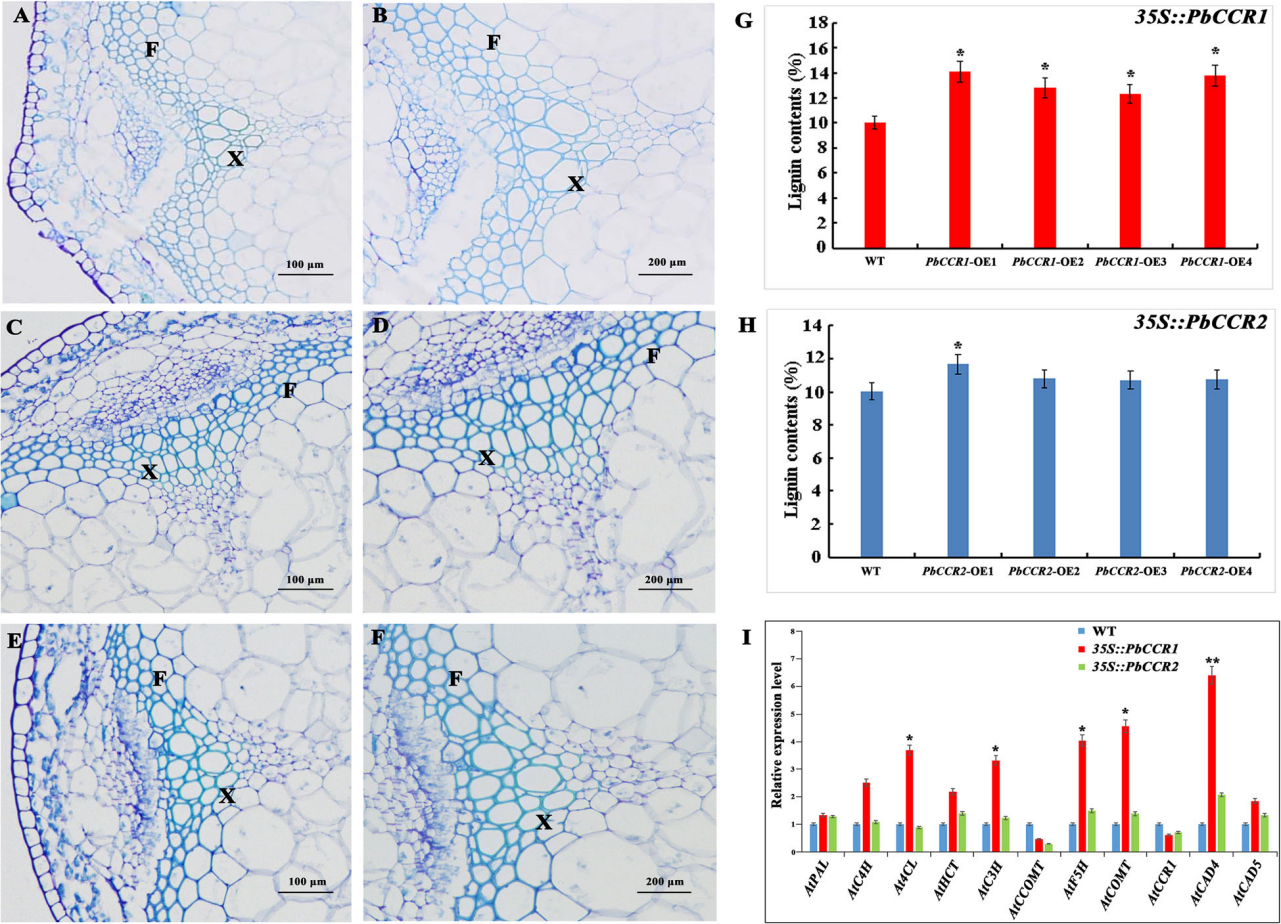
Observation of transverse section staining of inflorescence stems and determination of lignin contents in transgenic plants. **a**–**f** Toluidine blue staining of the inflorescence stems from wild-type (WT) and transgenic lines. **a**, **c**, **e**: bar = 100 μm. **b**, **d**, **f**: bar = 200 μm. F: interfascicular fibre; X: xylem. **g**–**h** Determination of lignin contents in inflorescence stems of Arabidopsis. Significant differences in lignin content levels between WT and transgenic overexpression lines of Arabidopsis were determined by a *t* test; *P < 0.05, **P < 0.01

**Fig. 8:**
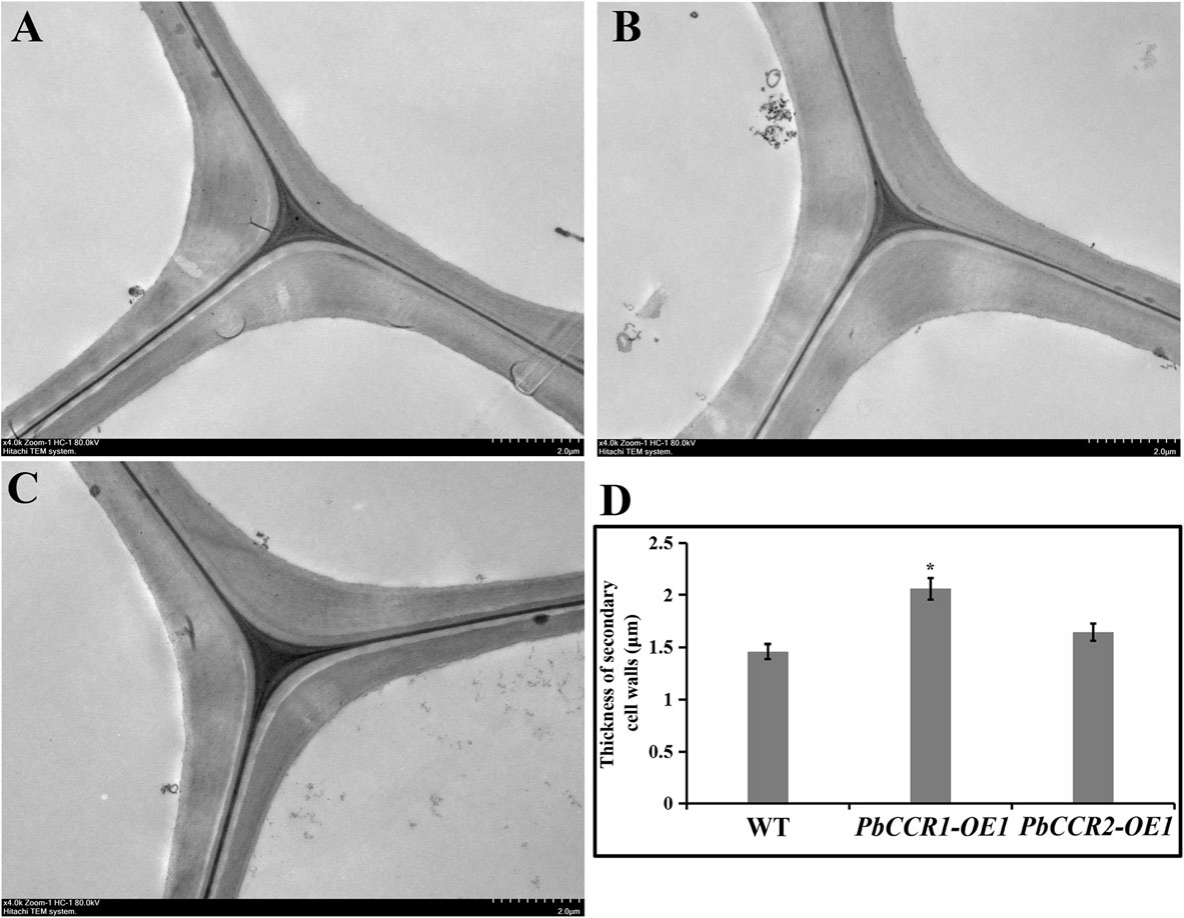
Micrographs of transmission electron microscopy on the cell wall of xylem of inflorescence stem of wild type and transgenic Arabidopsis (the scale = 2.0 μm). **a** Wild type; **b** *PbCCR1*-OE2; **c** *PbCCR2*-OE1; **d** Measurement of cell wall thickness (*P* < 0.05)

To understand the distribution of lignin in the inflorescence stems of wild-type and transgenic Arabidopsis more intuitively, we chose *PbCCR1*-OE2 and *PbCCR2*-OE1, which had higher lignin contents, and performed histochemical (toluidine blue) staining (Fig. 7a–f). We compared the stained cross-sections of the inflorescence stems of the three groups of samples. The staining intensities of the xylem and the interfascicular fibres of *PbCCR1*-OE2 were the highest, followed by those of *PbCCR2*-OE1, and those of wild-type plants were the lowest. In addition, we found that compared with wild-type plants, *PbCCR1*-OE2 showed an increase in the number of cells in the xylem region, while there was no significant difference between wild-type plants and *PbCCR2*-OE1. Subsequently, we observed the cell walls of the xylem in the cross-section of the inflorescence stem of Arabidopsis (wild type, *PbCCR1*-OE2 and *PbCCR2*-OE1) by transmission electron microscope (TEM) (Fig. 8a–c). The cell wall thickness of the xylem in the inflorescence stem cross-sections of *PbCCR1*-OE2 increased significantly compared with that of wild-type plants. In *PbCCR2*-OE1, the cell wall thickness of these cells also seemed to increase, but the change range was not as obvious as that observed in *PbCCR1*-OE2 plants (Fig. 8d).

We measured the lignin content in the inflorescence stems of Arabidopsis, which supported our staining results (Fig. 7g, h). The lignin contents of *PbCCR1*-OE2 and *PbCCR1*-OE4 reached 14.20 and 13.92%, respectively, which were significantly higher than those of wild-type plants (10.02%). However, although the lignin content (11.78%) of *PbCCR2*-OE1 was significantly higher than that of wild-type plants, and the lignin contents of the other lines were higher than that of wild-type plants, the difference in the other lines was not significant. In transgenic plants, the expression levels of key enzyme genes involved in Arabidopsis lignin biosynthesis changed greatly (Fig. 7i). Overexpression of *PbCCR1* in wild-type Arabidopsis increased the expression of most key lignin synthesis enzymes, especially *At4CL*, *AtF5H*, *AtCOMT*, *AtCAD4* and other genes. In contrast, the expression of *AtCCOMT* and *AtCCR1* was inhibited. However, in *PbCCR2*-OE1, the expression of many key lignin biosynthetic enzyme genes was also activated, but this activation was small. Similar to the results of *PbCCR1*-OE2, the expression of *AtCCOMT* and *AtCCR1* was inhibited.

## Discussion

The ‘Dangshan Su’ pear has a long history of cultivation and represents an important germplasm resources that occupies an important position in the world fruit market. However, in recent years, due to the deterioration of varieties and poor management, fruit quality has declined to some extent. The content of stone cells in pear fruit has increased, causing a less appealing taste. To solve this problem, many studies have been conducted on stone cells, and some progress has been made in revealing the biosynthetic and regulatory processes of pear stone cells and lignin [6, 39, 40]. However, the existing research has mainly concentrated on pear fruit after 15 DAP, and pear fruit in this period already contains a large number of stone cells [5, 41, 42]. The molecular mechanisms of stone cell formation and development in early fruit development (before 15 DAP) remain unclear. In this study, we selected fruit samples from the early developmental stages (0, 7, 15 DAP) of ‘Dangshan Su’ pear. We found that no stone cells were present near the fruit core at 0 DAP, indicating that the fruit did not form stone cell primordia during this period, and the metabolic pathways associated with stone cell formation and development may not be fully activated at this stage. At 7 DAP, sporadic stone cell primordia appeared near the core. At 15 DAP, the content of stone cells increased significantly (lignin content increased from 0.01 to 0.34%), and the metabolic network related to stone cell formation was fully activated (Fig. 1).

We then selected fruits obtained at 0, 7, 15 DAP for transcriptome sequencing to ascertain the molecular basis for the formation of large numbers of stone cells and lignin deposition at 0-15 DAP. We obtained a total of 35.2 Gb of data. For the 0, 7, 15 DAP fruit, we obtained at least 40506401, 25200858 and 32775750 mapped reads (Table 1), greater numbers than in the previous study of 23, 55 and 145 DAP fruit [5]. Because stone cells undergo a secondary wall thickening process accompanied by lignin deposition [43], we focused on cell wall development and lignin biosynthesis. The GO database contains three major categories, CC, MF, and BP, which can be applied to individual species to annotate the functions of genes [44]. For the 7-15 DAP comparison, in the BP classification (Additional file 18: Figure S10), a large number of DEGs were annotated to plant-type secondary cell wall biogenesis (44), phenylpropanoid biosynthetic process (44), lignin metabolic process (36), lignin biosynthetic process (32) and other functional categories. In the MF classification, many DEGs were annotated to GO nodes associated with plant cellulose biosynthesis, such as cellulose synthase activity (10) and cellulose synthase (UDP-forming) activity (10). All of the above are functional categories associated with cell wall development and lignin biosynthesis. However, in the 0-7 DAP period, where stone cells did not increase significantly, DEGs also fell into these functional categories, but the number of DEGs was significantly less than in the 7-15 DAP period. This finding indicated that the fruit began to produce stone cells on a large scale after 7 DAP.

The development of stone cells depends on the biosynthesis of lignin, which involves multiple metabolic pathways. The most important of these is the phenylpropanoid metabolic pathway (ko00360 and ko00940) [30]. Thirty-four key lignin biosynthetic enzyme genes were identified, and most of the genes showed an upward trend, such as *PbPAL1*, *Pb4CL1*, *2*, *PbHCT50*, *PbCCR1*, *2*, *PbCOMT1*, *3*, *PbPOD2* (Fig. 4, Table 2). These results showed that lignin biosynthesis entered an active stage after 7 DAP, and a large number of lignin monomers were synthesized. However, we found that there was no differential expression of *PbSAD* between 0 and 15 DAP, and this result was confirmed by qRT-PCR. SAD is involved in the late stage of lignin biosynthesis and can transform sinapaldehyde into sinapyl alcohol to produce syringyl monolignol [45]. In ‘Dangshan Su’ pear fruits at 23, 55 and 145 DAP, the expression of *PbSAD* was consistently very low. Perhaps the biosynthesis of syringyl monolignol in ‘Dangshan Su’ pear does not depend on the pathway of sinapyl alcohol catalysed by 4CL, CCR and SAD. The same results were also found in other species. For example, CAD may be involved in the synthesis of coniferyl alcohol and sinapyl alcohol in Arabidopsis and does not require any specific SAD to produce sinapyl alcohol [46]. A rice study also showed no evidence that a specific SAD is needed to produce sinapyl alcohol [47]. Therefore, we believe that the formation of syringyl monolignol in ‘Dangshan Su’ pear is mainly generated by caffeoyl-CoA catalysed by CCoAOMT, CCR, F5H and CAD. Among the genes involved in the polymerization of lignin monomers, the expression level of *PbLAC1* gradually increased. This gene has been shown to be involved in the biosynthesis of lignin and thickening of the cell wall [26]. In *PbPOD1*, *2*, *3*, only the expression level of *PbPOD2* (Pbr031894.1) increased as the lignin content increased. Therefore, we speculate that *PbLAC1* and *PbPOD2* are mainly responsible for the polymerization of lignin monomers in the fruit of ‘Dangshan Su’ pear. In addition to the phenylpropanoid pathway, we found that many metabolic pathways involved in lignin biosynthesis were up-regulated at 0-7 DAP. Many key enzyme genes (carbamoyl phosphate synthase, CPSase, Pbr026918.3; histidine phosphatase, His Phos, Pbr011138.1; D-isomer specific 2-hydroxyacid dehydrogenase, 2-Hacid dh, Pbr025526.1) in the carbon metabolic pathway (ko01200) are up-regulated during this period (Additional file 2: Table S2). The carbon metabolic pathway is activated to provide an important carbon source for downstream lignin biosynthesis [48]. At the same time, there are a number of up-regulated DEGs in the citrate cycle pathway (ko00020). Through the citrate cycle, sugars and other organic substances can be synthesized. These substances then enter glycolysis to form L-Phe [49]. This finding indicates that from 0 to 7 DAP, the fruit initiates upstream pathways to prepare for lignin biosynthesis, producing a large quantity of lignin biosynthetic precursors and accumulating materials for the large-scale synthesis of lignin after 7 DAP.

After 7 DAP, various pathways associated with lignin biosynthesis are activated, and the fruit begins to synthesize lignin in large quantities. At the same time, some metabolic pathways involved in the formation of plant cell walls also become active, for example, pentose and glucuronate interconversions (alcohol dehydrogenase GroES-like protein, ADH, Pbr013913.1; UDP-glucoronosyl and UDP-glucosyl transferase, UDPGT, Pbr009446.1; pentose phosphate pathway phosphofructokinase, PFK, Pbr008761.1) (Additional file 2: Table S2). The intermediates of these metabolic pathways are closely related to the biosynthesis of plant cell walls. In addition, we collected cellulose synthase genes from *Arabidopsis thaliana* and identified homologues of these genes in ‘Dangshan Su’ pear (Additional file 5: Table S5). According to previous studies, *AtCESA1*, *3*, *6* are mainly involved in the biosynthesis of the plant primary wall [50–52], while *AtCESA4*, *7*, *8* play an important role in the biosynthesis of plant secondary cell walls (SCWs) [53, 54]. We found that the homologous genes of *AtCESA4*, *7*, *8* (*PbCESA4-1*, *7*, *8*) in ‘Dangshan Su’ pear were up-regulated (Additional file 5: Table S5). To further verify that these three genes are involved in lignin biosynthesis in fruit stone cells, we explored the expression patterns of *PbCESA4-1*, *7*, *8* during the whole developmental period of ‘Dangshan Su’ pear (Additional file 23: Figure S15). The results showed that the expression patterns of these three genes were consistent with the developmental trends of lignin and stone cells in ‘Dangshan Su’ pear. This finding suggests that the three genes *PbCESA4-1*, *7*, *8* may be involved in the development of stone cells in pear fruit and may be responsible for SCW formation.

We investigated the expression patterns of MYB, NAC and AP2/ERF transcription factors in ‘Dangshan Su’ pear from 0 to 15 DAP to identify genes closely related to stone cell formation (Additional file 22: Figure S14). Arabidopsis NST1, 2, 3 have been shown to regulate SCW formation in plants [32, 55]. We identified the NAC gene most closely related to *AtNST1*, *2*, *3* in ‘Dangshan Su’ pear and found that only the *AtNST1* homologous gene (*PbNAC46*) showed up-regulated expression at 0-15 DAP (Additional file 6: Table S6). Thus, we believe that *PbNAC46* is the most important NAC gene involved in SCW formation in ‘Dangshan Su’ pear stone cells. MYB transcription factors can induce, catalyse and hinder the biosynthesis of plant lignin [56]. In this study, we found that multiple MYB genes may be involved in the development of pear stone cells (Additional file 6: Table S6). We found that the homologous genes of *AtMYB46* and *83* (functional redundancy, positive regulation of secondary wall, cellulose, lignin biosynthesis) in ‘Dangshan Su’ pear (*PbMYB105*, *104*) were up-regulated, suggesting that there may be a regulatory network similar to that of Arabidopsis in ‘Dangshan Su’ pear. This regulatory mechanism is achieved through the regulation of downstream genes by *PbMYB105* and *104* [57]. In addition, we identified several MYB genes that may be involved in the regulation of lignin biosynthesis (including the activation or inhibition of lignin biosynthesis), such as *PbMYB103*, *122*, *127* (Additional file 6: Table S6). Compared with NAC and MYB transcription factors, AP2/ERF genes often regulate lignin biosynthesis by directly regulating the expression of key enzyme genes in lignin biosynthesis [58–60]. We found that the expression of *PbSHN* (Pbr027839.1) is consistent with the accumulation of lignin content, and we believe this gene may have similar functions to *AtSHN* in regulating the biosynthesis of lignin [33]. *PbERF110* has very close affinities with some ERF genes that regulate lignin biosynthesis (*PtERF34*, *DcERF2*, *OsERF71*) [60–62]. We believe that *PbERF110* is a potential AP2/ERF gene that positively regulates lignin biosynthesis in ‘Dangshan Su’ pear. Conversely, *PbAP2-24* was discovered as a possible negative regulation of lignin biosynthetic genes. We found that the similarity between the amino acid sequences of *PbAP2-24* and *EjAP2-1* (a transcription factor that inhibits lignin biosynthesis) [63] was as high as 96.75% (Additional file 24: Figure S16). Both the transcriptome sequencing and qRT-PCR results showed that *PbAP2-24* was consistently low from 0 to 15 DAP. This finding indicates that *PbAP2-24* did not exert its biological function of inhibiting lignin synthesis during the activation period of stone cell development and lignin synthesis.

CCR, as a key enzyme gene regulating lignin monomer biosynthesis, plays an important role in the lignin biosynthetic pathway. There has been controversy regarding the types of substrates used by CCR genes in catalytic reactions, but numerous studies have shown that CCR is more likely to use feruloyl-CoA or caffeoyl-CoA rather than sinapoyl-CoA as a substrate in catalytic reactions [64]. In this study, we do not believe that the biosynthesis of S-type lignin in ‘Dangshan Su’ pear depends on the catalytic activity of *PbSAD*. The reaction of sinapoyl-CoA catalysed by *PbCCR* is located upstream of the reaction, which indicates that *PbCCR* in ‘Dangshan Su’ pear probably does not use sinapoyl-CoA as the substrate for catalytic reaction (Fig. 4). Thirty-one CCR genes were retained in the ‘Dangshan Su’ pear genome, and *PbCCR1*, *2*, and *3* were likely to participate in lignin biosynthesis [24]. According to the results of the study of expression profiles, only *PbCCR1* and *2* showed up-regulated expression patterns, and the expression level of *PbCCR3* in this period was very low (Fig. 5a). We believe that *PbCCR1* and *2* may be closely related to early stone cell development and lignin biosynthesis in pear fruit, while *PbCCR3* has little effect on stone cell development and lignin biosynthesis in the early fruit development stage. To further clarify the roles of *PbCCR1* and *2* in lignin biosynthesis, we overexpressed these two genes in wild Arabidopsis (Fig. 6). The functions of *PbCCR1* and *2* were verified by overexpression analysis. We found that overexpression of *PbCCR1* increased lignin content in transgenic plants and cell wall thickness in xylem tissues and interfascicular fibres (Fig. 8). Similar results have been reported in birch, where overexpression of *BpCCR1* increased the lignin content of transgenic plants to 14.6% and increased the cell wall thickness of xylem vessel cells [65]. However, overexpression of *PbCCR2* did not play a significant role in these indicators, perhaps because *PbCCR2* showed low catalytic activity, so it could not cause significant changes in lignin content. This finding may be observed because overexpression of *PbCCR1* activates the expression of most lignin biosynthetic genes in Arabidopsis, but the activation resulting from *PbCCR2* overexpression is weak (Fig. 7i). Overall, *PbCCR1* and *2* have a degree of functional redundancy, and both demonstrate the ability to participate in lignin biosynthesis. However, *PbCCR1* may be the major gene for lignin biosynthesis, while *PbCCR2* has little effect on lignin biosynthesis. This finding is consistent with the fact that there are multiple CCR genes with catalytic ability in a species but only one major gene [38, 66].

## Conclusion

By investigating pear fruit at 0, 7 and 15 DAP, we found that primordial stone cells began to appear at 7 DAP, and the fruit had formed a large number of stone cells at 15 DAP. We performed transcriptome sequencing on the fruits at 0, 7 and 15 DAP and identified 3834 (0 vs. 7 DAP), 4049 (7 vs. 15 DAP) and 5763 (0 vs. 15 DAP) DEGs. The stone cells of ‘Dangshan Su’ pear fruit began to form in large quantities after 7 DAP, and the key enzyme genes in the lignin biosynthetic pathway were up-regulated widely. Before 7 DAP, the fruit primarily activates upstream material metabolic pathways (carbon metabolism and citrate cycle pathway) to provide important precursor substances for lignin biosynthesis. Further analysis found that the biosynthesis of S-type lignin in ‘Dangshan Su’ pear does not depend on the catalytic activity of *PbSAD* but is primarily generated by the catalytic activity of caffeoyl-CoA through CCoAOMT, CCR, F5H and CAD. In addition, some transcription factors regulating lignin biosynthesis were identified, such as *PbNAC46*, *PbMYB105*, *104*, *PbSHN* and *PbAP2-24*. Subsequently, we cloned *PbCCR1* and *2* and analysed their functions in the Chinese white pear lignin biosynthetic process. Overall, *PbCCR1*, *2* has a degree of functional redundancy, both of which demonstrate the ability to participate in lignin biosynthesis. However, *PbCCR1* may be the major gene for lignin biosynthesis, while *PbCCR2* has little effect on lignin biosynthesis.

## Methods

### Plant materials

The plant material used in this experiment was the ‘Dangshan Su’ pear, which grows in the Yuanyichang agricultural park (Dangshan County, Anhui Province, China). In the flowering period of ‘Dangshan Su’ pear (approximately April 1 of each year), ‘Cuiguan’ (*Pyrus pyrifolia*) was used as the pollen parent of the fruit. We first collected the pollen of ‘Cuiguan’ for pollinating ‘Dangshan Su’ pear. Selecting 40-year-old *Pyrus bretschneideri* cv. Dangshan Su trees with good growth, the stamens of the flowers were manually removed, and the pollen was applied to the stigma for artificial pollination. Immediately after artificial pollination, the stigma was wrapped in a bag and cultured for 7 days in the dark. After artificial pollination, we ensure that the fruits after pollination were kept under the same growth conditions, including water and fertilizer and management conditions. At 7 and 15 DAP, we collected the fruit for transcriptome sequencing along with the fruit from 0 DAP. For consistency with our previous study of the stone cells and lignin of ‘Dangshan Su’ pear, we collected the corresponding fruits for qRT-PCR analysis at 39, 47, 55, 63, 79, 102, and 145 DAP. Some of the 0, 7, and 15 DAP fresh fruit was used for sectioning, microscopic observation and determination of stone cell and lignin contents, and the remaining fruits were stored in a − 80 °C freezer for subsequent experiments. Three random biological repeats were set up for fruit collection in each period to eliminate the errors caused by differences between fruit samples.

### Determination of stone cells and lignin in pear fruit

The fruits at 0, 7, and 15 DAP were selected for the determination of stone cell and lignin contents. Each group of samples was weighed to 5.0 g of fruit and frozen at − 20 °C for 1 day. The fruit samples were then homogenized at 20000 r·min^− 1^ for 5 min. Water was added to the homogenate, which was then allowed to stand. After all of the contents sank to the bottom of the beaker, the suspended matter in the upper layer was poured out (this operation was repeated five times). Finally, the stone cells were dried and weighed, and the stone cell content of each fruit sample was calculated as follows: formula weight of stone cells (g DW)/weight of pulp (g FW) × 100 = stone cell content (%) [5].

The lignin content of ‘Dangshan Su’ pear fruit was also determined at 0, 7, and 15 DAP. The fruit epidermis was removed and dried in an oven at 37 °C, and the dried fruit was ground finely. The powder was extracted with methanol after grinding, and then, the extracted residue was dried. Next, 0.2 g dried residue was extracted for 1 h in 15 ml 70% H_2_SO_4_ at 30 °C. After extraction, 115 mL of distilled water was added, and the solution was boiled for 1 h; the total volume of the sample remained unchanged during boiling. The boiled mixture was filtered with filter paper and rinsed with 70 °C water until the rinse water was clear. The lignin residue was dried and weighed, and each sample was repeated three times.

### Extraction of total RNA from plant material and qRT-PCR

The total RNA of all the used ‘Dangshan Su’ pear fruits in this study was extracted using a plant RNA extraction kit from Tiangen (Beijing, China) (Additional file 9: Figure S1). Reverse transcription after total RNA extraction from the fruit was performed using a PrimeScript™ RT reagent kit with gDNA Eraser (Takara, Kusatsu, Japan). All qRT-PCR primers in this study were designed using Beacon Designer 7 software, and liquid primers were ordered from Sangon Biotech (Shanghai, China) (Additional file 7: Table S7). We tested the amplification efficiency of all primers by reference to the method of Zhu et al. (Additional file 7: Table S7) [67]. At the same time, the standard curves of each primer in the detection of amplification efficiency were listed (Additional file 25: Figure S17). The qRT-PCR system consisted of 10 μL SYBR® Premix Ex TaqTM II (2X), 6.4 μL water, 0.8 μL upstream and downstream primers and 2 μL cDNA (20 μL total). For the qRT-PCR experiment, we chose the tubulin gene (No. AB239680.1) as the internal reference gene [68]. Each gene was evaluated with three biological replicates during qRT-PCR, and the 2^-△△CT^ method was used to process the data and calculate the relative expression levels of the genes [69].

### Transcriptome sequencing

Referring to the methods of total RNA extraction and reverse transcription in plants, a DNA library of fruit samples from 0, 7, 15 DAP stages was constructed. Three sets of biological replicates were set for each period of fruit (a total of 9 cDNA libraries were constructed for transcriptome sequencing) to eliminate errors caused by differences between fruits. The constructed library was analysed using the Illumina HiSeq™ 2500 sequencing platform (Sangon Biotech, Shanghai, China). From the raw data, we first filtered out the adaptor sequences and low-quality reads. The remaining sequences were considered clean reads and were mapped to the reference genome of Chinese white pear using HISAT2 software. Finally, unigene expression was calculated as FPKM with the software package Cufflinks [70]. The raw data has been uploaded to the SRA database of NCBI (Additional file 1: Table S1).

### Identification, classification and metabolic pathway analysis of differentially expressed genes

In this experiment, either 0 DAP was used as the control and 7, 15 DAP were used as the experimental groups or 7 DAP was used as the control and 15 DAP was used as the experimental group. Differential expression analysis was performed to find genes with different expression levels between the two samples. A direct expression of a gene’s expression level is the abundance of its transcript. The higher the transcript abundance, the higher the gene expression level is. We used the TPM value to calculate the expression level of each gene. The BlastX and BlastN programs were used to compare the assembled unigenes with protein and nucleic acid databases (e-value< 10^− 5^). These databases include the nonredundant protein database (http://ncbi.nlm.nih.gov/), the nonredundant nucleic acid sequence database (http://ncbi.nlm.nih.gov/), the KEGG database (http://www.kegg.jp), the Cluster of Orthologous Groups (COG) database (https://www.ncbi.nlm.nih.gov/COG/), and the GO annotation database (http://www.geneontology.org). GO annotations, including MF, BP and CC, were also analysed. The best alignment results were selected for functional annotation of the gene and to determine the metabolic pathway in which the gene is involved.

### Cloning of genes and construction of plant expression vectors

Full-length sequence-specific primers were designed using Primer Premier 6.0 software based on the full-length sequences of *PbCCR1*, *2*. RT-PCR was performed using ‘Dangshan Su’ pear fruit cDNA as template. Primers with restriction sites were also designed using Primer Premier 6.0 software (*PbCCR1* and *2* both used NcoI and SpeI restriction endonuclease sites) (Additional file 8: Table S8). Each gene fragment was ligated into the pCAMBIA1304 vector using T4 ligase at 16 °C for 3 h to obtain complete pCAMBIA1304-*PbCCR1*, *2* recombinant plasmids.

### Screening of transgenic Arabidopsis and determination of lignin content

Enrichment culture of Agrobacterium liquid carrying pCAMBIA1304-*PbCCR1* and *2* plasmids was performed. After the enrichment culture was completed, the supernatant was discarded after centrifugation, and the cell pellet was retained. The cells were resuspended in invasion buffer (0.02% Silwet L-77, 1/2 MS, 5% sucrose) such that the OD600 value of the solution was between 0.7 and 0.8 for subsequent infection. Wild-type Arabidopsis plants with a large number of flower buds and good growth condition were selected for infection (Arabidopsis seeds were purchased from the Arabidopsis Biological Resource Center). The whole flower buds were soaked in the Agrobacterium solution for approximately 45 s. After infection, the Arabidopsis plants were cultured in a dark environment for 24 h and then moved out of the dark environment and grown under normal light. The seeds were collected after maturation, sterilised, and germinated on MS medium containing hygromycin. The plants that could grow normally were potential positive plants, and the positive transgenic plants were obtained after a GUS staining test.

According to the results of Anderson et al., the acetyl bromide method was used to detect lignin content [71]. The inflorescence stems of the 60-day-old T3 generation of transgenic Arabidopsis were sliced by hand, stained with 1% toluidine blue on slides and observed directly under a microscope. TEM analysis was performed with reference to the method of Wu et al. [72].

## Supplementary information

**Additional file 1: Table S1.** Accession number of RNA-Seq raw data in NCBI SRA database.

**Additional file 2: Table S2.** Summary of KEGG pathways.

**Additional file 3: Table S3.** KEGG pathway classification.

**Additional file 4: Table S4.** The expression level of putative novel genes related to stone cell development.

**Additional file 5: Table S5.** Cellulose synthase gene related to secondary wall biosynthesis in Chinese white pear.

**Additional file 6: Table S6.** Putative functions of pear MYB, NAC, AP2/ERF transcription factors.

**Additional file 7: Table S7.** Sequence of primers for qRT-PCR.

**Additional file 8: Table S8.** Primer sequences used in transgenic Arabidopsis.

**Additional file 9: Figure S1.** RNA quality testing of all nine samples.

**Additional file 10: Figure S2.** PCA analysis of biological repeats of 0, 7, 15 DAP samples.

**Additional file 11: Figure S3.** Duplicate correlation checking scatter plot analysis of biological repeats of 0, 7, 15 DAP samples.

**Additional file 12: Figure S4.** Read distribution in different regions blasted against the reference genome.

**Additional file 13: Figure S5.** Relationship between gene TPM deviation within 15% of the final value (based on 100% mapped reads) and percentage of mapped reads.

**Additional file 14: Figure S6.** Density distribution of gene expression in 0, 7, 15 DAP fruit. Gene expression levels are represented as log_2_(TPM).

**Additional file 15: Figure S7.** Cluster analysis of transcript expression.

**Additional file 16: Figure S8.** Volcano plot of differentially expressed genes.

**Additional file 17: Figure S9.** Unigene KOG function categories.

**Additional file 18: Figure S10.** Unigene GO function categories.

**Additional file 19: Figure S11.** Visual KEGG path classification.

**Additional file 20: Figure S12.** DEGs related to phenylpropanoid metabolism in the 7 DAP vs. 15 DAP comparison. Red indicates upregulation of expression, while yellow indicates no change in expression.

**Additional file 21: Figure S13.** qRT-PCR validation of the expression levels of putative novel genes related to stone cell development.

**Additional file 22: Figure S14.** Expression profiles of the MYB, NAC, and AP2/ERF genes in Chinese white pear fruit at 0, 7, and 15 DAP. The fragments per kilobase of exon per million mapped reads (TPM) values were obtained by RNA sequencing analysis and are presented as a heat map. The colour scale shows different levels of gene expression. Red indicates a high level of expression, white signifies a medium level of expression, and blue denotes a low level of expression.

**Additional file 23: Figure S15.** Expression patterns of *PbCESA4-1*, *7*, *8* in fruit at different developmental stages.

**Additional file 24: Figure S16.** *PbAP2-24* alignment with *EjAP2-1* proteins.

**Additional file 25: Figure S17.** Standard curve of qRT-PCR primers.

## Data Availability

Data supporting the results of this study can be obtained from [NCBI], but the availability of these data is limited (accession number: SRR9998933, SRR9998932, SRR9998940, SRR9998936, SRR9998935, SRR9998934, SRR9998939, SRR9998938, SRR9998937). These data are used under the permission of the current study and are therefore not publicly available. We chose to publish our data on June 20, 2020. Until such a time, the data is available from the corresponding author upon reasonable request.

## Abbreviations

4CL: 4-hydroxycinnamate-CoA ligase
BP: Biological process
C4H: Cinnamate 4-hydroxylase
CAD: Cinnamyl alcohol dehydrogenase
CC: Cellular component
CCoAOMT: Caffeoyl-CoA O-methyltransferase
CCR: Cinnamoyl-CoA reductase
*CESA*: Cellulose synthase
COMT: Caffeic acid 3-O-methyltransferase
DAP: Days after pollination
DEGs: Differentially expressed genes
F5H: Ferulate-5-Hydroxylase
GO: Gene ontology
KEGG: Kyoto encyclopedia of genes and genomes
KOG: Karyotic ortholog groups
LAC: Laccase
L-Phe: L-phenylalanine
MF: Molecular function
PAL: Phenylalanine ammonia-lyase
POD: Peroxidase
SAD: Sinapyl alcohol dehydrogenase
SCW: Secondary cell wall
SDRs: Short-chain dehydrogenase superfamilies
TPM: Transcripts per million
