# Supplementary material for: Transcriptomic analysis of early fruit development in Chinese white pear (Pyrus bretschneideri Rehd.) and functional identification of PbCCR1 in lignin biosynthesis
Source: BMC Plant Biol. 2019 Oct 11;19:417. doi: 10.1186/s12870-019-2046-x (PMC6788021; doi:10.1186/s12870-019-2046-x)
Supplement: Supplementary file 1 — Additional file 1: Table S1. Accession number of RNA-Seq raw data in NCBI SRA database. [file 12870_2019_2046_MOESM1_ESM.docx]

| **Number** | **Sample name** | **Accession number** |
| --- | --- | --- |
| **1** | **'Dangshan Su' pear fruit for 0 days-1** | **SRR9998933** |
| **2** | **'Dangshan Su' pear fruit for 0 days-2** | **SRR9998932** |
| **3** | **'Dangshan Su' pear fruit for 0 days-3** | **SRR9998940** |
| **4** | **'Dangshan Su' pear fruit for 7 days-1** | **SRR9998936** |
| **5** | **'Dangshan Su' pear fruit for 7 days-2** | **SRR9998935** |
| **6** | **'Dangshan Su' pear fruit for 7 days-3** | **SRR9998934** |
| **7** | **'Dangshan Su' pear fruit for 15 days-1** | **SRR9998939** |
| **8** | **'Dangshan Su' pear fruit for 15 days-2** | **SRR9998938** |
| **9** | **'Dangshan Su' pear fruit for 15 days-3** | **SRR9998937** |

**Table S1.** Accession number of RNA-Seq raw data in NCBI SRA Database.
