# Supplementary material for: Transcriptomic analysis of early fruit development in Chinese white pear (Pyrus bretschneideri Rehd.) and functional identification of PbCCR1 in lignin biosynthesis
Source: BMC Plant Biol. 2019 Oct 11;19:417. doi: 10.1186/s12870-019-2046-x (PMC6788021; doi:10.1186/s12870-019-2046-x)
Supplement: Supplementary file 4 — Additional file 4: Table S4. The expression level of putative novel genes related to stone cell development. [file 12870_2019_2046_MOESM4_ESM.docx]

**Table S4.** The expression level of putative novel genes related to stone cell development.

| **Gene**  **name** | **Genome ID** | **0 DPA VS 7 DPA TPM** | | | **7 DPA VS 15 DPA TPM** | | | | **0 DPA VS 15 DPA TPM** | | |
| --- | --- | --- | --- | --- | --- | --- | --- | --- | --- | --- | --- |
|  |  | **0 DPA** | **7 DPA** | **DEGs** | **7 DPA** | **15 DPA** | **DEGs** | **0 DPA** | | **15 DPA** | **DEGs** |
| ***DIR*** | **Pbr000712.1** | **7.4867** | **40.940** | **UP** | **40.940** | **118.375** | **UP** | **7.4867** | | **118.375** | **UP** |
| ***KIP1*** | **Pbr001414.1** | **1.736** | **8.256** | **UP** | **8.256** | **21.833** | **UP** | **1.736** | | **21.833** | **UP** |
| ***S-AdoMet*** | **Pbr001686.1** | **20.551** | **53.004** | **UP** | **53.001** | **360.508** | **UP** | **20.551** | | **360.508** | **UP** |
| ***GATase*** | **Pbr002524.1** | **2.735** | **5.581** | **UP** | **5.581** | **12.011** | **UP** | **2.735** | | **12.011** | **UP** |
| ***GMC oxred*** | **Pbr011059.2** | **1.217** | **9.954** | **UP** | **9.954** | **32.330** | **UP** | **1.217** | | **32.330** | **UP** |
| ***EamA*** | **Pbr024589.1** | **0.499** | **6.238** | **UP** | **6.238** | **13.831** | **UP** | **0.499** | | **13.831** | **UP** |
| ***TPS*** | **Pbr024829.1** | **0.465** | **8.547** | **UP** | **8.547** | **39.377** | **UP** | **0.465** | | **39.377** | **UP** |
| ***Cu-oxidase*** | **Pbr025074.1** | **2.006** | **9.460** | **UP** | **9.460** | **21.649** | **UP** | **2.006** | | **21.649** | **UP** |
