# Supplementary material for: Transcriptomic analysis of early fruit development in Chinese white pear (Pyrus bretschneideri Rehd.) and functional identification of PbCCR1 in lignin biosynthesis
Source: BMC Plant Biol. 2019 Oct 11;19:417. doi: 10.1186/s12870-019-2046-x (PMC6788021; doi:10.1186/s12870-019-2046-x)
Supplement: Supplementary file 5 — Additional file 5: Table S5. Cellulose synthase gene related to secondary wall biosynthesis in Chinese white pear. [file 12870_2019_2046_MOESM5_ESM.docx]

| *AtCESA* | Cellulose synthase gene in Chinese white pear | | | | | DGEs |
| --- | --- | --- | --- | --- | --- | --- |
|  | *PbCESA* | Genome ID | 0 DAP TPM | 7 DAP TPM | 15 DAP TPM |  |
| *AtCESA1* | *PbCESA1* | Pbr027819.1 | 117.968 | 113.433 | 180.797 | * |
| *AtCESA2* | *PbCESA2-1* | Pbr007542.1 | 10.615 | 4.024 | 12.503 | * |
|  | *PbCESA2-2* | Pbr023836.1 | 56.252 | 64.753 | 93.182 | * |
| *AtCESA3* | *PbCESA3-2* | Pbr004913.1 | 49.359 | 62.618 | 100.026 | UP |
| *AtCESA4* | *PbCESA4-1* | Pbr013444.1 | 27.161 | 36.297 | 55.226 | UP |
|  | *PbCESA4-2* | Pbr039970.1 | 62.345 | 62.401 | 50.567 | * |
| *AtCESA5* | *PbCESA5* | Pbr030463.1 | 33.876 | 44.889 | 123.601 | UP |
| *AtCESA6* | *PbCESA6* | Pbr019484.1 | 51.708 | 47.729 | 166.796 | UP |
| *AtCESA7* | *PbCESA7* | Pbr034219.1 | 1.443 | 4.151 | 112.019 | UP |
| *AtCESA8* | *PbCESA8* | Pbr000518.1 | 0.237 | 1.733 | 27.855 | UP |
| *AtCESA9* | *PbCESA9* | Pbr015135.1 | 0.067 | 0.039 | 0.0001 | * |
| *AtCESA10* | *PbCESA10* | Pbr002623.1 | 76.129 | 76.651 | 141.223 | * |

**Table S5.** Cellulose synthase gene related to secondary wall biosynthesis in Chinese white pear.
