# Supplementary material for: Transcriptomic analysis of early fruit development in Chinese white pear (Pyrus bretschneideri Rehd.) and functional identification of PbCCR1 in lignin biosynthesis
Source: BMC Plant Biol. 2019 Oct 11;19:417. doi: 10.1186/s12870-019-2046-x (PMC6788021; doi:10.1186/s12870-019-2046-x)
Supplement: Supplementary file 6 — Additional file 6: Table S6. Putative functions of pear MYB, NAC, AP2/ERF transcription factors. [file 12870_2019_2046_MOESM6_ESM.docx]

**Table S6.** Putative functions of pear MYB, NAC, AP2/ERF transcription factors.

| **Gene name** | **Gene ID** | **Differential expression** | ***A .thaliana* and**  **other plants** | **Functions** | **References** |
| --- | --- | --- | --- | --- | --- |
| **MYB gene family** | |  |  |  |  |
| *PbMYB8* | Pbr038922.1 | Down |  |  |  |
| *PbMYB22* | Pbr024420.1 | Down | *AtMYB57* |  |  |
| *PbMYB27* | Pbr028812.1 | Down | *AtMYB62* | Stress responses | Abe et al. (2003); Devaiah et al. (2009) |
| *PbMYB35* | Pbr030940.1 | UP | *AtPHL62* | Regulate plant transcriptional response to Pi starvation | Sun et al. (2016) |
| *PbMYB37* | Pbr011095.1 | UP | *AtMYB15* | Involved in cold stress tolerance | Agarwal et al. (2006) Reyes and Chua. (2007) |
| *PbMYB44* | Pbr041525.1 | UP | *AtMYB60* | Respond to environmental  stress | Raffaele et al. (2008) Seo and Park. (2009) |
| *PbMYB47* | Pbr014381.1 | Down and UP | *AtMYB32* |  |  |
| *PbMYB58* | Pbr011441.1 | UP | *AtMYB31* |  |  |
| *PbMYB66* | Pbr039864.1 | UP | *AtMYB55* |  |  |
| *PbMYB72* | Pbr001888.1 | UP | *AtMYB86* |  |  |
| *PbMYB73* | Pbr032528.1 | Down and UP | *AtMYB96* |  |  |
| *PbMYB80* | Pbr020733.1 | Down |  |  |  |
| *PbMYB82* | Pbr038922.1 | Down |  |  |  |
| *PbMYB83* | Pbr020726.1 | Down |  |  |  |
| *PbMYB91* | Pbr019293.1 | UP |  |  |  |
| *PbMYB93* | Pbr006685.1 | Down and UP | *AtMYB61* |  |  |
| *PbMYB94* | Pbr005982.2 | UP | *AtMYB50* |  |  |
| *PbMYB103* | Pbr036591.1 | UP | *PtMYB4* | Regulation of secondary wall biosynthesis | Zhong et al. (2011) |
| *PbMYB104* | Pbr036590.1 | UP | *AtMYB83* | Regulation of secondary wall, cellulose and lignin biosynthesis | Zhong et al. (2011) |
| *PbMYB105* | Pbr017813.1 | UP | *AtMYB46* | Regulation of secondary wall, cellulose and lignin biosynthesis | Zhong et al. (2011) |
| *PbMYB106* | Pbr012310.1 | UP |  |  |  |
| *PbMYB107* | Pbr008748.1 | UP |  |  |  |
| *PbMYB109* | Pbr024492.1 | UP |  |  |  |
| *PbMYB122* | Pbr038701.2 | UP |  |  |  |
| *PbMYB127* | Pbr028319.1 | UP |  |  |  |
| *PbMYB131* | Pbr027035.1 | Down |  |  |  |
| *PbMYB132* | Pbr019687.1 | Down |  |  |  |
| **NAC gene family** | |  |  |  |  |
| *PbNAC9* | Pbr024863.1 | UP |  |  |  |
| *PbNAC10* | Pbr024864.1 | Down |  |  |  |
| *PbNAC39* | Pbr000412.1 | UP |  |  |  |
| *PbNAC46* | Pbr038584.1 | UP | *AtNST1* | Regulation of lignin biosynthesis | Mitsuda et al., 2005; Yang et al., 2007 |
| *PbNAC74* | Pbr025898.1 | UP |  |  |  |
| *PbNAC87* | Pbr022333.1 | UP |  |  |  |
| *PbNAC119* | Pbr027119.1 | UP |  |  |  |
| *PbNAC145* | Pbr038436.1 | Down |  |  |  |
| *PbNAC155* | Pbr038565.1 | Down | *AtNAC56* |  |  |
| *PbNAC170* | Pbr004500.1 | Down | *AtNAC83* |  |  |
| *PbNAC172* | Pbr032231.1 | Down |  |  |  |
| *PbNAC173* | Pbr032232.1 | Down |  |  |  |
| **AP2/ERF gene family** | |  |  |  |  |
| *PbERF1* | Pbr015697.1 | UP |  |  |  |
| *PbERF5* | Pbr027423.1 | UP |  |  |  |
| *PbERF6* | Pbr018367.1 | UP |  |  |  |
| *PbERF8* | Pbr009571.1 | UP |  |  |  |
| *PbERF35* | Pbr025302.1 | UP |  |  |  |
| *PbERF39* | Pbr000396.1 | UP |  |  |  |
| *PbERF61* | Pbr010949.1 | UP |  |  |  |
| *PbERF67* | Pbr016049.2 | UP |  |  |  |
| *PbERF102* | Pbr001363.1 | UP |  |  |  |
| *PbERF110* | Pbr012024.1 | UP | *PtERF34, DcERF2,*  *OsERF71* | Regulate lignin biosynthesis | Jorma et al., 2013; Soichi et al., 2008; Lee et al., 2016 |
| *PbERF126* | Pbr027839.1 | UP | *AtSHN2* | Regulate lignin biosynthesis | Ambavaram et al., 2011 |
| *PbERF127* | Pbr015502.1 | UP | *AtERF35* |  |  |
| *PbERF132* | Pbr017391.1 | Down |  |  |  |
| *PbAP2-5* | Pbr001083.1 | UP |  |  |  |
| *PbAP2-11* | Pbr015227.1 | UP |  |  |  |
| *PbrAP2-22* | Pbr019788.1 | UP |  |  |  |
| *PbrAP2-24* | Pbr015291.2 | * | *EjAP2-1* | Regulate lignin biosynthesis | Zeng et al., 2015 |
