# Supplementary material for: Transcriptomic analysis of early fruit development in Chinese white pear (Pyrus bretschneideri Rehd.) and functional identification of PbCCR1 in lignin biosynthesis
Source: BMC Plant Biol. 2019 Oct 11;19:417. doi: 10.1186/s12870-019-2046-x (PMC6788021; doi:10.1186/s12870-019-2046-x)
Supplement: Supplementary file 7 — Additional file 7: Table S7. Sequence of primers for qRT-PCR. [file 12870_2019_2046_MOESM7_ESM.docx]

**Table S7.** Sequence of primers for qRT-PCR.

| Gene name | Primer sequences 5’ | Primer sequences 3’ | Amplification efficiency |
| --- | --- | --- | --- |
| *AtPAL* | AAGATTGGAGCTTTCGAGGA | TCTGTTCCAAGCTCTTCCCT | 86.9% |
| *AtC4H* | ACTGGCTTCAAGTCGGAGAT | ACACGACGTTTCTCGTTCTG | 89.1% |
| *At4CL* | TCAACCCGGTGAGATTTGTA | TCGTCATCGATCAATCCAAT | 94.7% |
| *AtHCT* | GCCTGCACCAAGTATGAAGA | GACAGTGTTCCCATCCTCCT | 94.2% |
| *AtC3H* | GTTGGACTTGACCGGATCTT | ATTAGAGGCGTTGGAGGATG | 110.1% |
| *AtCCOMT* | CTCAGGGAAGTGACAGCAAA | GTGGCGAGAAGAGAGTAGCC | 74.7% |
| *AtF5H* | CTTCAACGTAGCGGATTTCA | AGATCATTACGGGCCTTCAC | 96.3% |
| *AtCOMT* | TTCCATTGCTGCTCTTTGTC | CATGGTGATTGTGGAATGGT | 91.8% |
| *AtCCR1* | GTGCAAAGCAGATCTTCAGG | GCCGCAGCATTAATTACAAA | 94.9% |
| *AtCAD4* | CACTTCGGTCTAATGGCGAGT | AGTCAAGGGAATCTGCGAGTCT | 73.7% |
| *AtCAD5* | TTGGCTGATTCGTTGGATTA | ATCACTTTCCTCCCAAGCAT | 76.5% |
| *β-tubulin4* | GGAGCTACGCAGAACAACTAAGA | CCCACGAGGATCATAGTTGCAACTGA | 70.5% |
| *PbPAL1* | CGCCACTTATCCATTGATG | CTTCTCACTCTCGCCATT | 86.2% |
| *PbPAL2* | CGAGAATTGAAGACAGTT | ACTTATACAACGGATAGGA | 112.1% |
| *PbPAL3* | TTAGATTCTTGAACGCTGGA | CTCTTGTTGCTTGGTGTG | 83.7% |
| *PbC4H1* | AACGACTTCAGGTATCTC | ATTGTAATGCCCAGGATT | 94.2% |
| *PbC4H2* | TGAACCATCCTGAAATCC | GCTCTGTGATCTGAACTC | 95.6% |
| *PbC4H3* | CTCAAGGCACAGCAGAAG | TTCAATAGCAGCGGCATT | 62.2% |
| *PbC3H* | TCAAGCCTGCCTTACAAT | GACGAGGGAACAGTAGTG | 96.7% |
| *Pb4CL1* | ACATCAAACAATACATCTCA | CTTTGCTCTCAAGTCTTT | 81.1% |
| *Pb4CL2* | CGACAAGGACAACCATAG | GCTCATCATCATCATCAAGA | 103.2% |
| *PbCCoAOMT* | CAATTCTTGAACATGCTTCTC | GGAGTAGCCAGTGTAGAC | 79.2% |
| *PbHCT2* | AGACCCTCCTTTACTGGCAACATAT | TTATAAGTGTACTAACCGCAGTTAA | 72.6% |
| *PbHCT17* | ATACCACATTGTGTAGCTGATGGAG | TGCAGAGGGGTTTTTTTAGGG | 72.4% |
| *PbHCT18* | CTGTCACGGCTGATTCTC | GATTTGATATGCTCGGATT | 140.8% |
| *PbHCT49* | GTTAGCGTGAGGGAGTCAACA | GGTCGAAGAACGAGTTGCCA | 77.5% |
| *PbHCT50* | CAACGAAGCGCGAAGATGGT | CTCGGAATCACCAGGTCCAC | 88.5% |
| *PbF5H1* | AACAATAGACTCGCTAAG | TCTCACCATCATTCAATC | 169.6% |
| *PbF5H2* | AACAATAGACTCGCTAAG | TACCATTCCTCTTCTTCT | 97.9% |
| *PbCCR1* | AGAGCAGAATCATACAAGTTCACAA | TTTTATAAGCAAGATCTAATTTTAA | 109.2% |
| *PbCCR2* | CGTCCGGAAACAAAGCTAATAAACA | GGTTCCTCTAACAGTGTAGCCTCTTTCG | 75.5% |
| *PbCCR3* | CTAATAACCAAGATGCCTGCCGATA | GGTTCCTCTAACAGTGTAGCCTCTTTCG | 118.7% |
| *PbCOMT1* | TACATCAACTACCACAAGAG | CACAGACCCATTCCATAG | 88.4% |
| *PbCOMT3* | TATATTGGAGACGAGTGT | AGAAGCATGTTCAAGAAT | 118.0% |
| *PbCAD1* | GTAGAGATTGCGACTATTGT | TTGTAATCGTGCCATCAG | 102.3% |
| *PbCAD2* | AAGGAAACTGAGGAGATGCTTGAAT | TACTTTATTAAATAAGATTGCTGCCG | 128.4% |
| *PbCAD3* | GAGTAGGAGATGTGGTAG | GTATTGCTCATTGTCTGT | 149.6% |
| *PbLAC1* | TGGCTTTTCTTCTTGCTCTTATCTC | CATTGCTGCAGCTGGCAGT | 116.3% |
| *PbLAC14* | AGCCAATGTGCCCAAAACTG | GGCATGGGTTGGTTCCAA | 113.8% |
| *PbPOD1* | CCTTCCACAAGATTCTGAA | GATATTCTCGTCTTGTCTG | 89.5% |
| *PbPOD2* | GATATTCTCGTCTTGTCTG | TCCTTCCATCTCTTCTTC | 74.1% |
| *PbPOD3* | AACTTCCAACTTCAGCAATC | GAGCACCAGATAGCAAGA | 89.5% |
| *PbSAD* | GCGGAGGTTGAAGTTATC | TCGGTATCTTACATCATTCTTAG | 201.1% |
| *DIR* | CCAACCAATTCATCAACAG | GAGGCAGACGAATAGAAC | 95.3% |
| *KIP1* | ATCGTTCTACTTCTATGTG | ATTCCTCAACCAATTCAG | 103.8% |
| *S-AdoMet* | ATGATGAGACTGTCACAA | GGTTAAGGTGGAAGATTG | 135.8% |
| *GATase* | CAGATATGAGGTTAATCC | CTAAGAATAGAGCAGAAG | 98.9% |
| *EamA* | CTCTCACCATTGTTATCT | AAGCAACTGTATCAAGAA | 98.3% |
| *TPS* | ATAACTACATACCAACCAT | GAGACTTCATATCATCCA | 103.0% |
| *GMC oxred* | CAATTCTTGCTCAATCTG | ATTCGTATAACTCATTCATC | 78.6% |
| *Cu-oxidase* | AGGCATTACAGCAATCTT | TTCGCATTCAACTAACTTC | 99.4% |
| *PbCESA4-1* | CCTCATTGATTCGATTCC | CTCAGTCTTGTCTTCGTA | 88.4% |
| *PbCESA7* | TCCTCTTCTTGCCTATTG | GTTCCAAAGATTGAGAGAAA | 127.2% |
| *PbCESA8* | TACGGTCCTCCTTCTATG | CTCGGTAAGCCTCTGATA | 100.7% |
