# Supplementary material for: Transcriptomic analysis of early fruit development in Chinese white pear (Pyrus bretschneideri Rehd.) and functional identification of PbCCR1 in lignin biosynthesis
Source: BMC Plant Biol. 2019 Oct 11;19:417. doi: 10.1186/s12870-019-2046-x (PMC6788021; doi:10.1186/s12870-019-2046-x)
Supplement: Supplementary file 8 — Additional file 8: Table S8. Primer sequences used in transgenic Arabidopsis. [file 12870_2019_2046_MOESM8_ESM.docx]

**Table S8.** Primer sequences used in transgenic Arabidopsis.

| **Gene name** | **Primer sequences 5’** | **Primer sequences 3’** |
| --- | --- | --- |
| ***PbCCR1*** | **ATGCCTGCCGTTAGCTCGTCAG** | **AGATTGAATTTTAATGGAATCTTG** |
| ***PbCCR2*** | **ATGCCTGCCGATAGCTCATCA** | **TTATAAGCAAGATCTAAT** |
| ***PbCCR1-1304*** | **CCCATGGGG ATGCCTGCCG** | **CTAGACTAGT AGATTGAATTT** |
| ***PbCCR2-1304*** | **CCCATGGGGATGCCTGCCGAT** | **CTAGACTAGTTAAGCAAGATCTAATTTT** |
