# Supplementary figures and images for: Transcriptomic analysis of early fruit development in Chinese white pear (Pyrus bretschneideri Rehd.) and functional identification of PbCCR1 in lignin biosynthesis
Source: BMC Plant Biol. 2019 Oct 11;19:417. doi: 10.1186/s12870-019-2046-x (PMC6788021; doi:10.1186/s12870-019-2046-x)

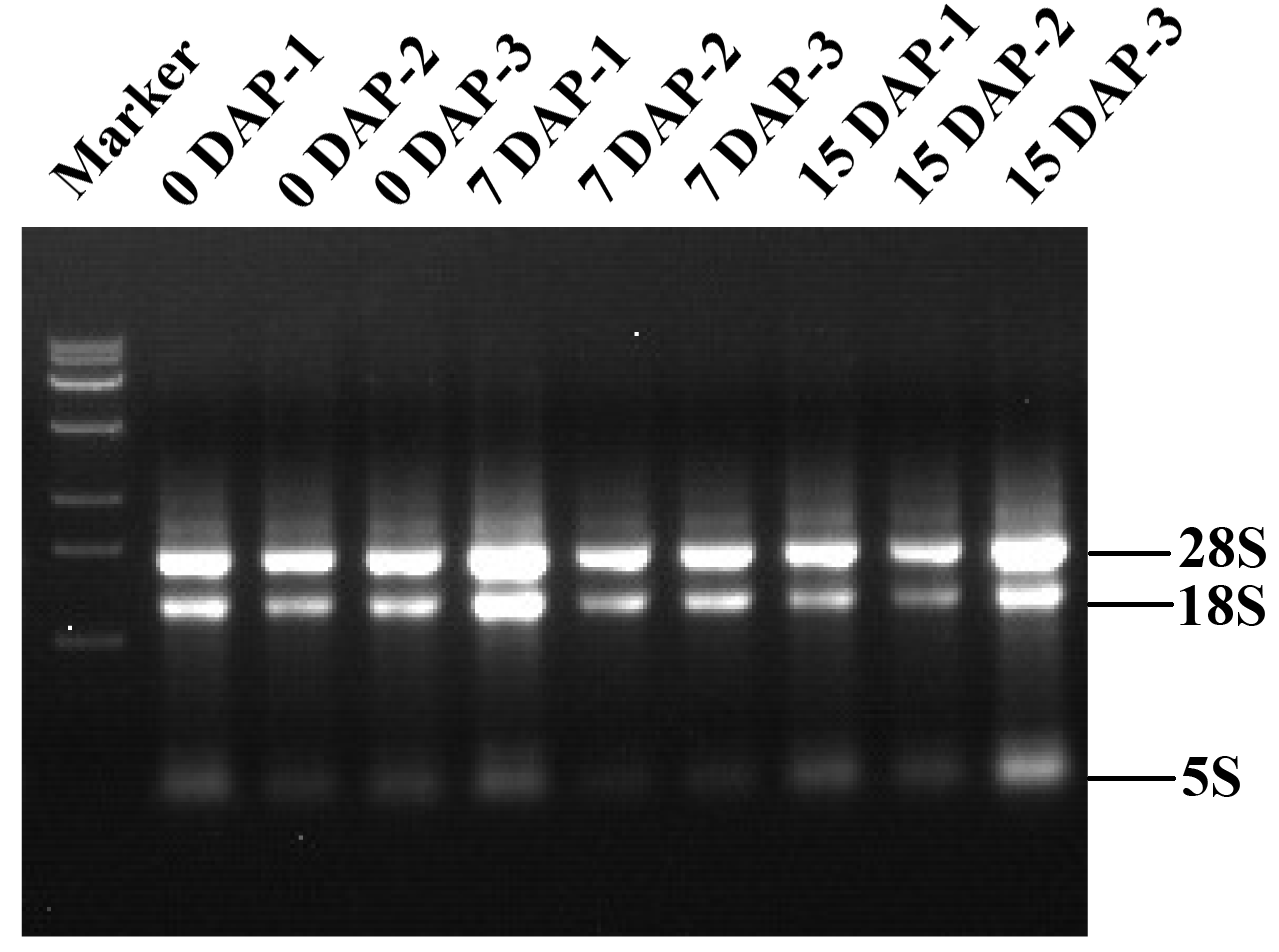

Supplement: Supplementary file 9 — Additional file 9: Figure S1. RNA quality testing of all nine samples. [file 12870_2019_2046_MOESM9_ESM.tiff]

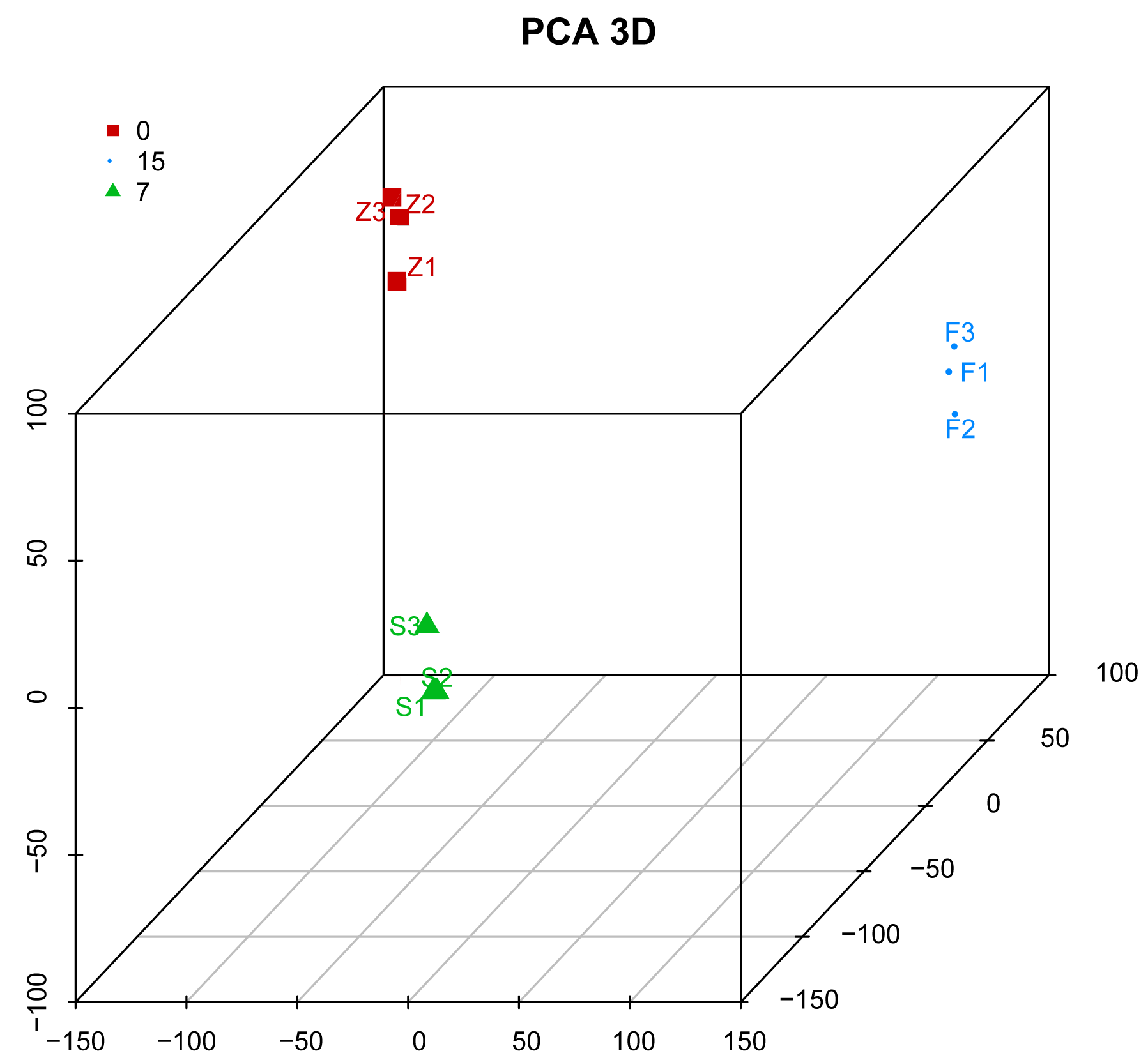

Supplement: Supplementary file 10 — Additional file 10: Figure S2. PCA analysis of biological repeats of 0, 7, 15 DAP samples. [file 12870_2019_2046_MOESM10_ESM.tiff]

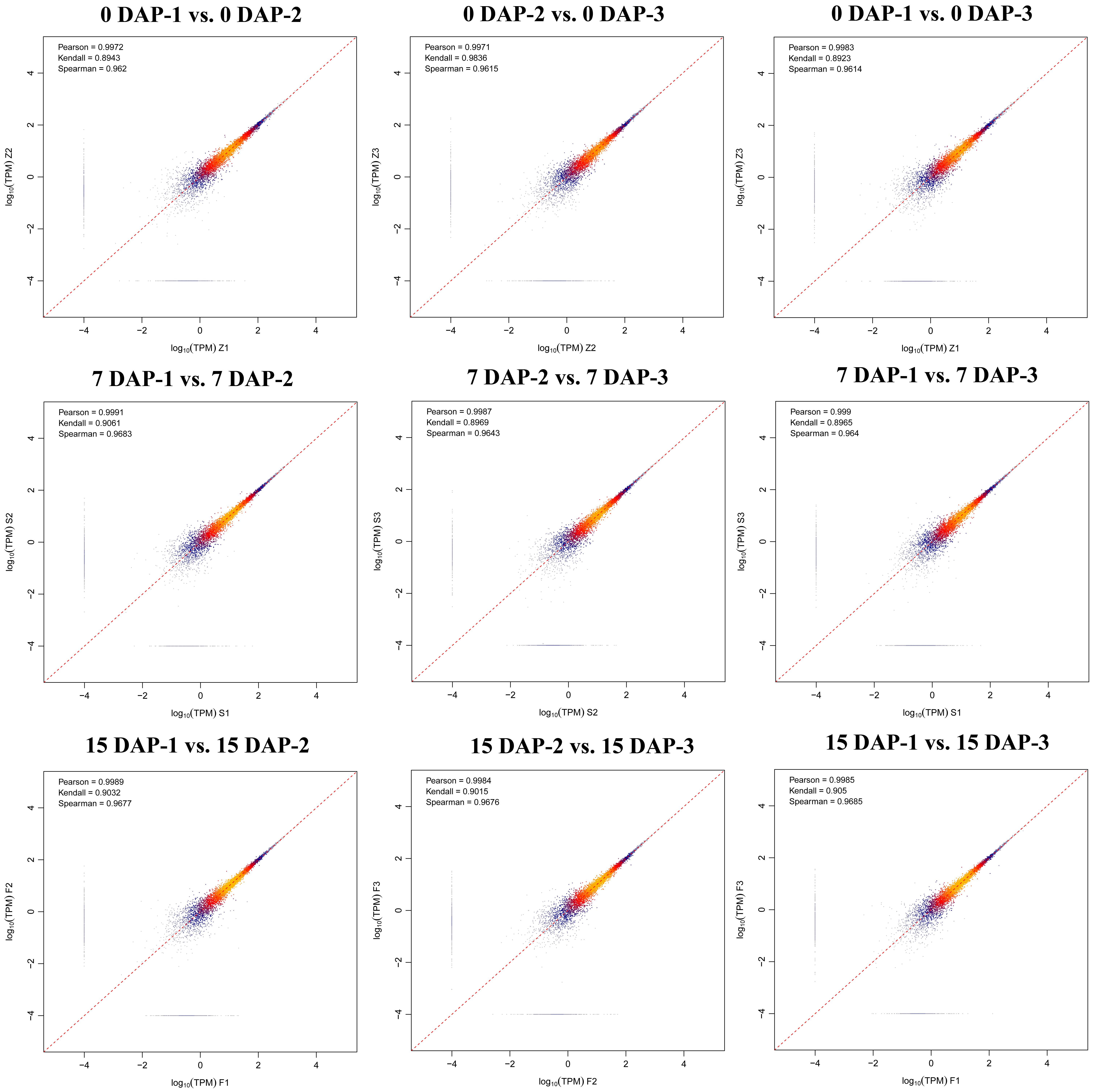

Supplement: Supplementary file 11 — Additional file 11: Figure S3. Duplicate correlation checking scatter plot analysis of biological repeats of 0, 7, 15 DAP samples. [file 12870_2019_2046_MOESM11_ESM.tiff]

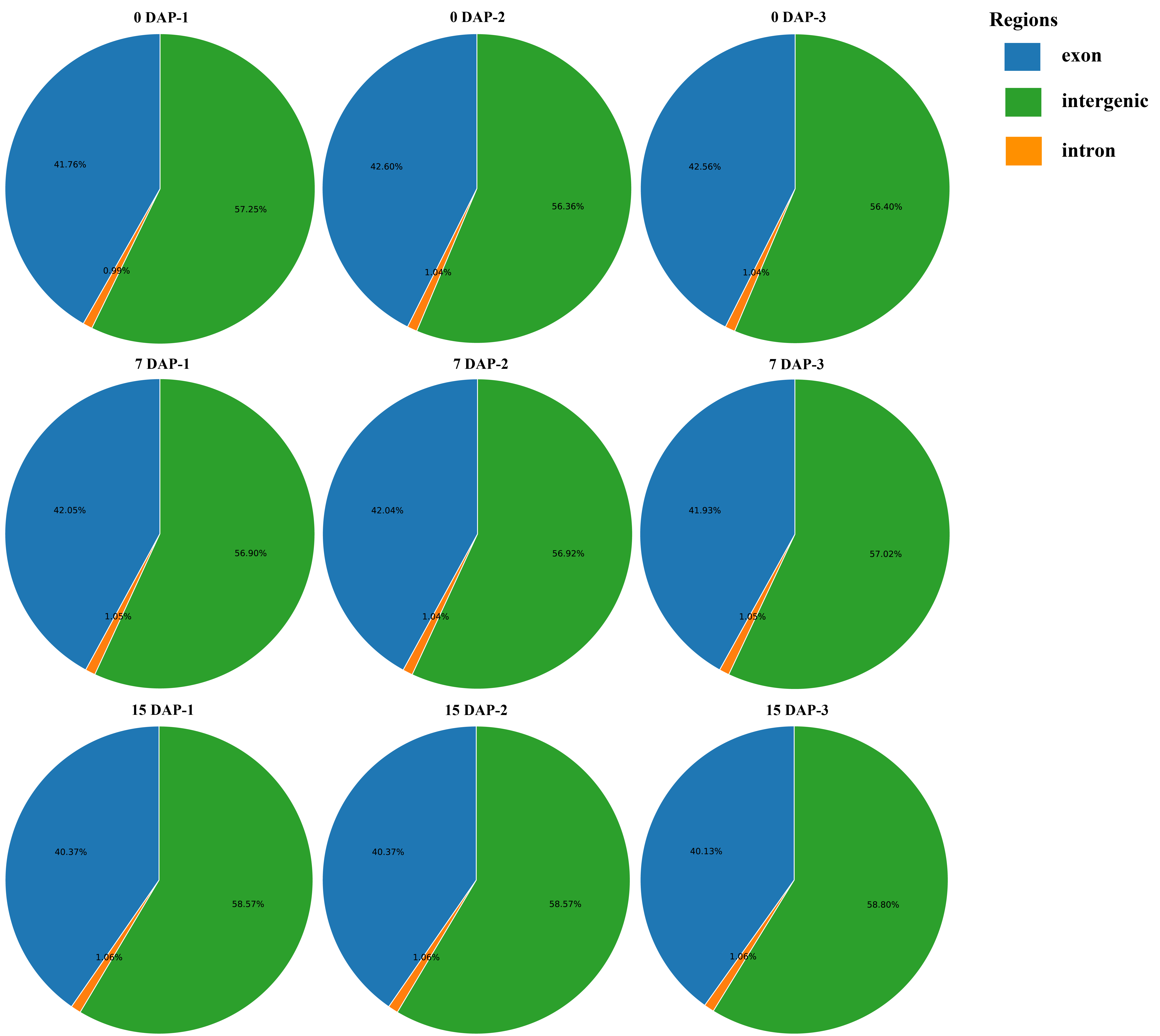

Supplement: Supplementary file 12 — Additional file 12: Figure S4. Read distribution in different regions blasted against the reference genome. [file 12870_2019_2046_MOESM12_ESM.tiff]

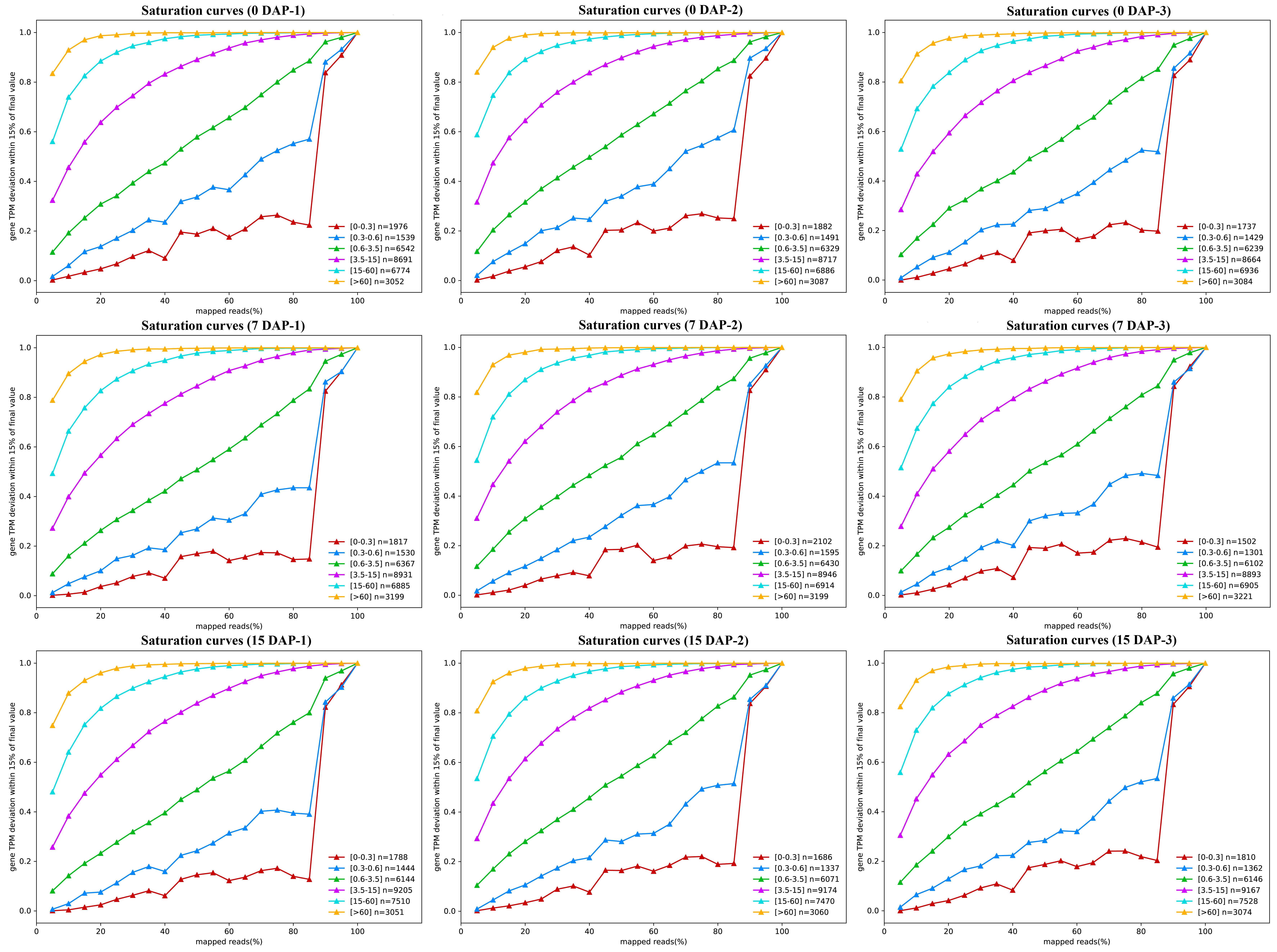

Supplement: Supplementary file 13 — Additional file 13: Figure S5. Relationship between gene TPM deviation within 15% of the final value (based on 100% mapped reads) and percentage of mapped reads. [file 12870_2019_2046_MOESM13_ESM.tiff]

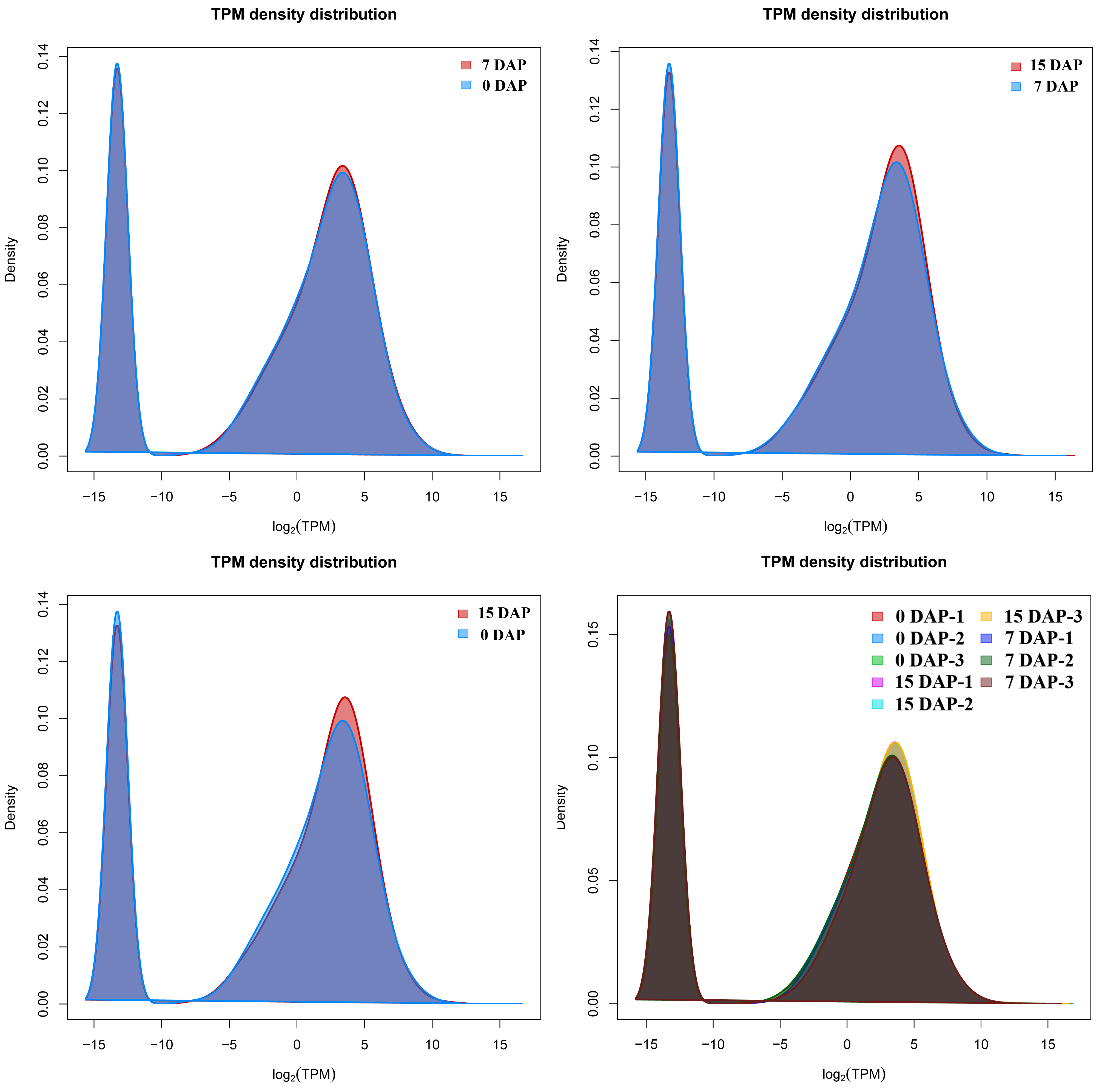

Supplement: Supplementary file 14 — Additional file 14: Figure S6. Density distribution of gene expression in 0, 7, 15 DAP fruit. Gene expression levels are represented as log2(TPM). [file 12870_2019_2046_MOESM14_ESM.tiff]

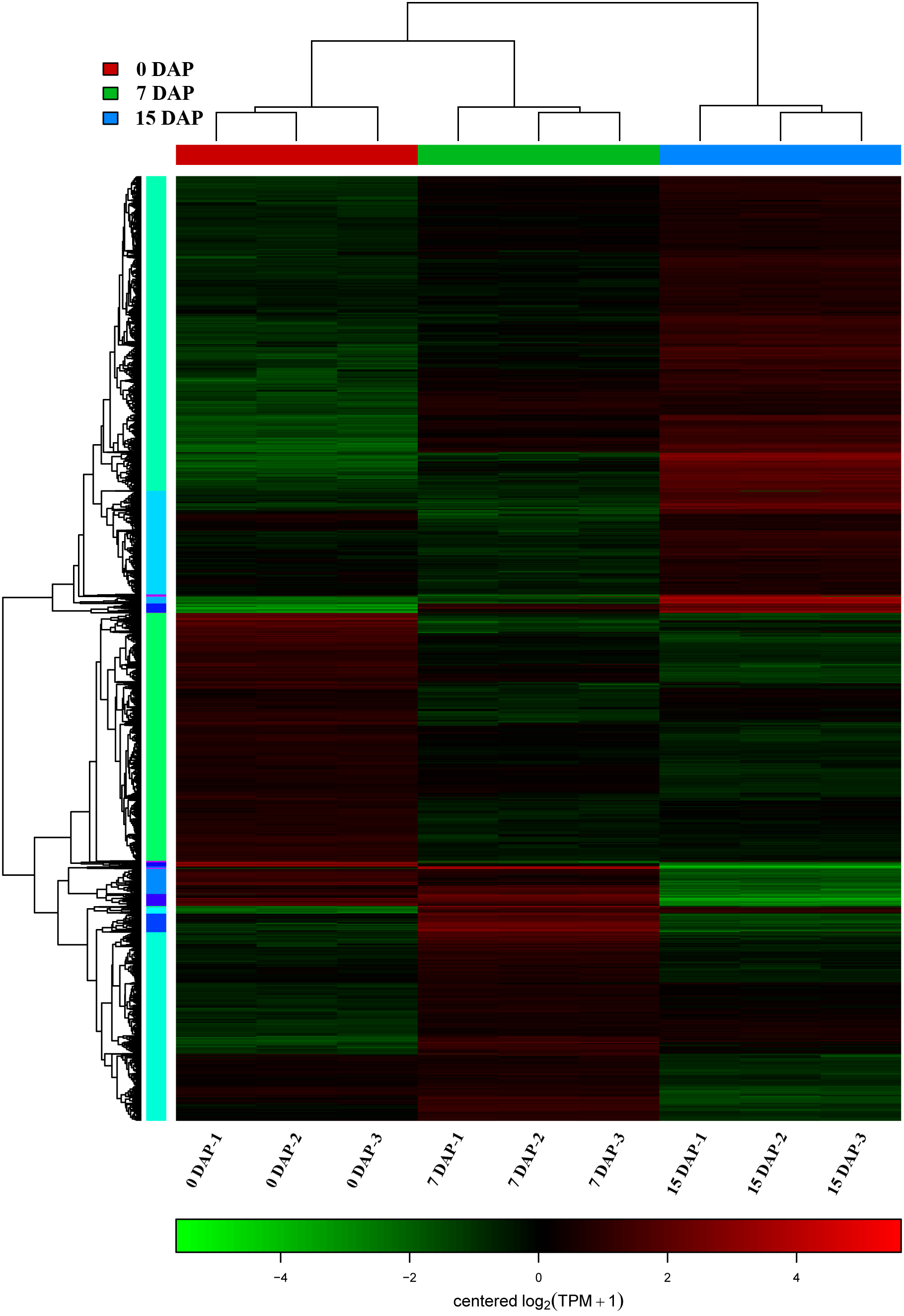

Supplement: Supplementary file 15 — Additional file 15: Figure S7. Cluster analysis of transcript expression. [file 12870_2019_2046_MOESM15_ESM.tiff]

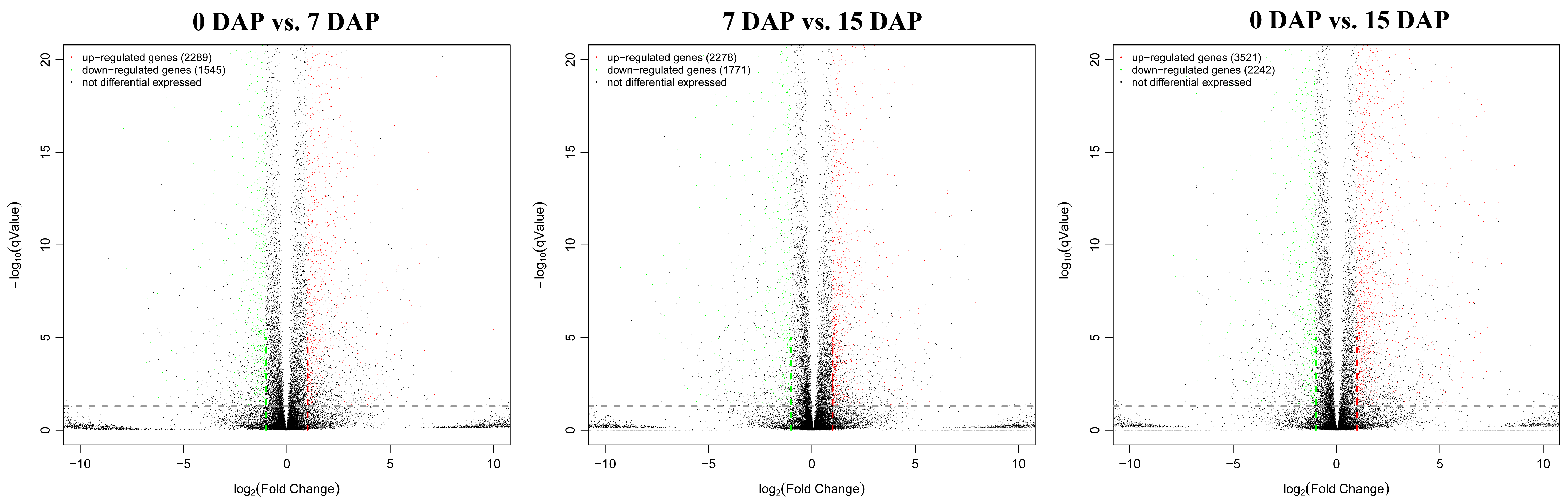

Supplement: Supplementary file 16 — Additional file 16: Figure S8. Volcano plot of differentially expressed genes. [file 12870_2019_2046_MOESM16_ESM.tiff]

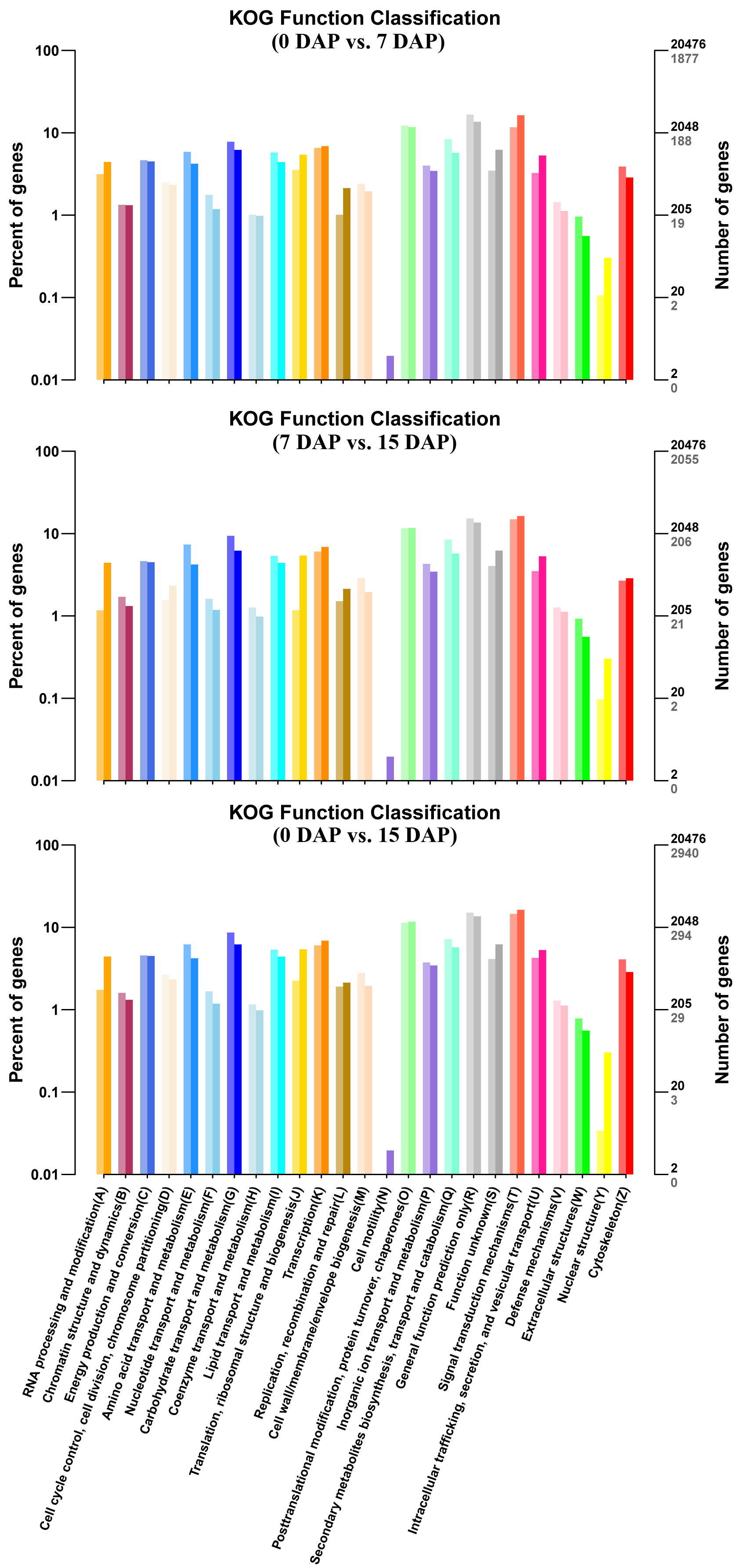

Supplement: Supplementary file 17 — Additional file 17: Figure S9. Unigene KOG function categories. [file 12870_2019_2046_MOESM17_ESM.tiff]

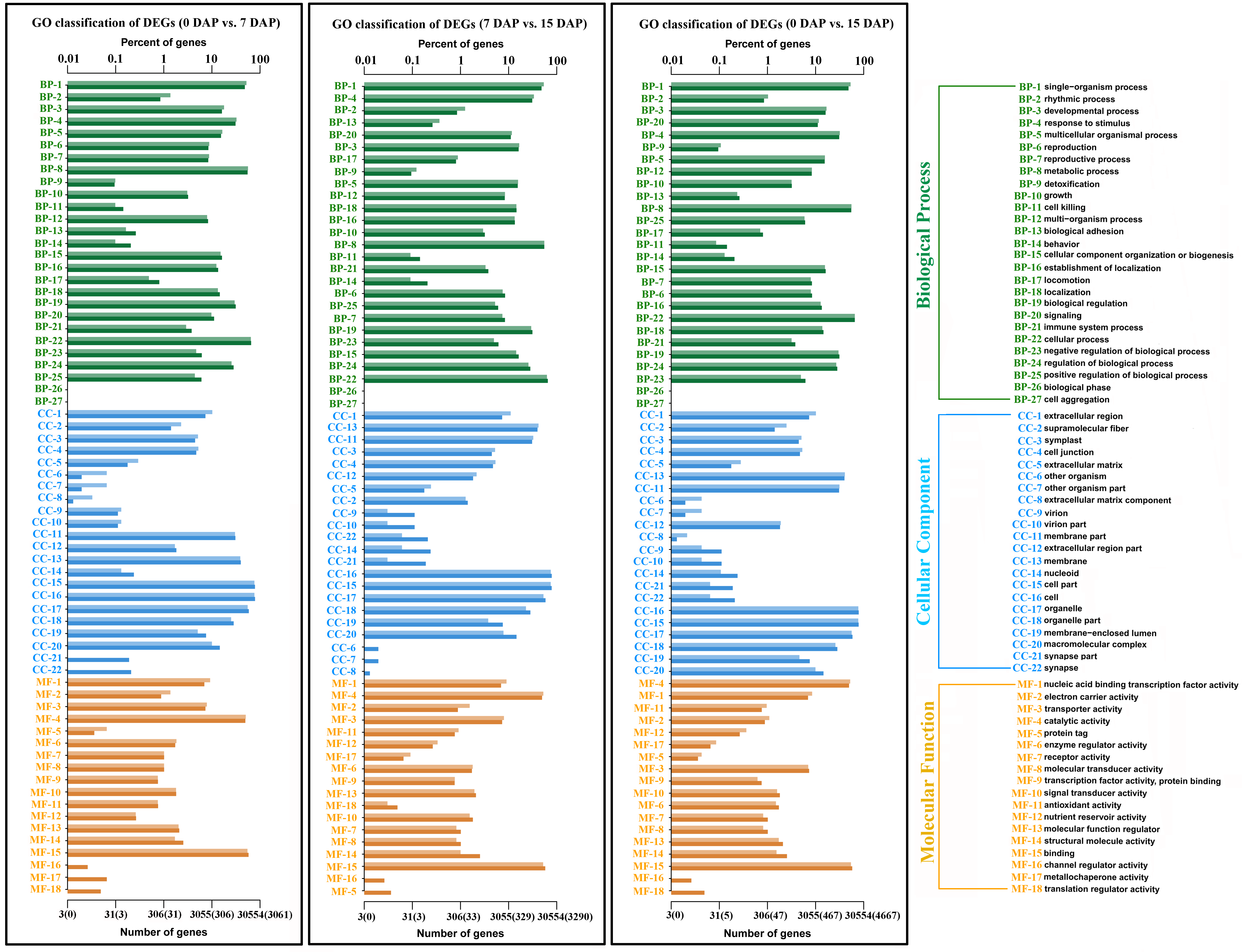

Supplement: Supplementary file 18 — Additional file 18: Figure S10. Unigene GO function categories. [file 12870_2019_2046_MOESM18_ESM.tiff]

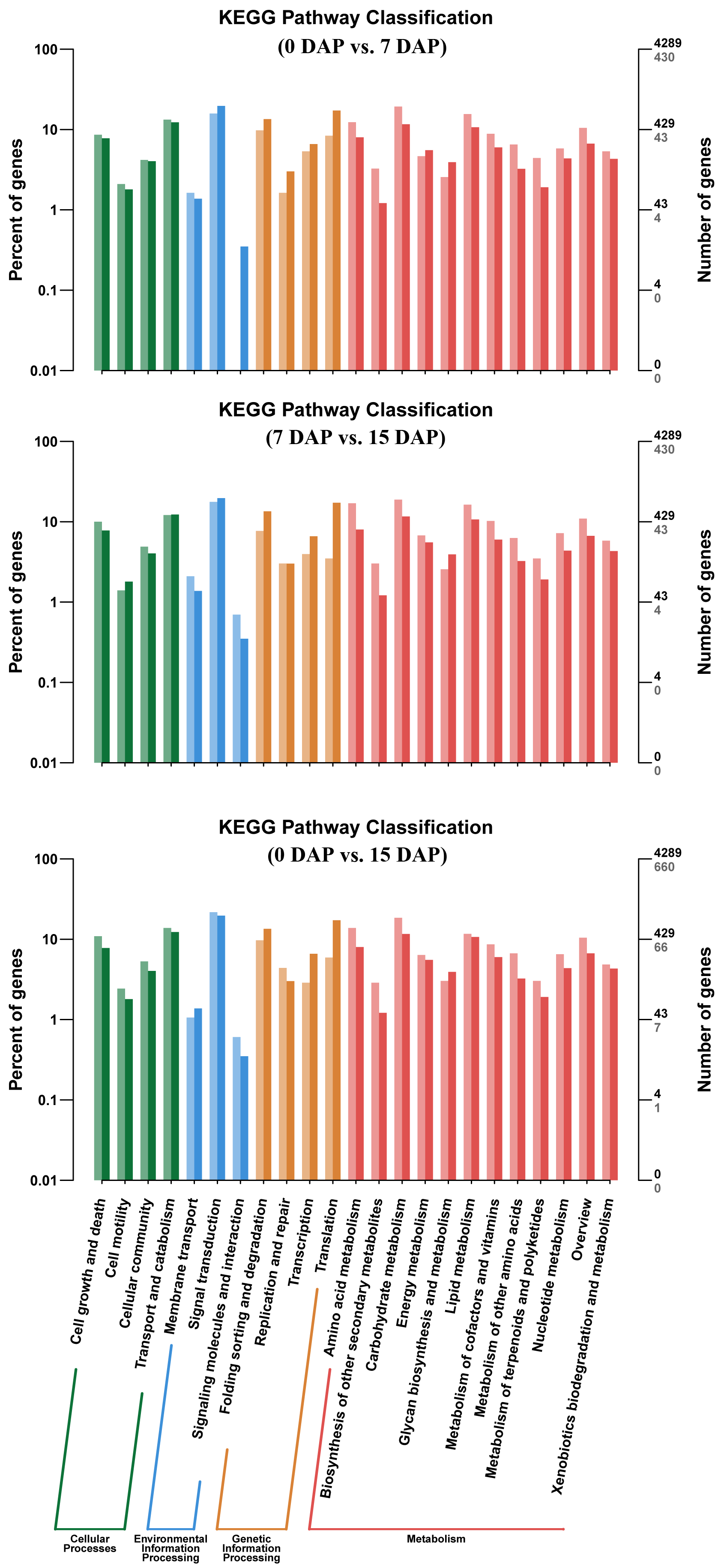

Supplement: Supplementary file 19 — Additional file 19: Figure S11. Visual KEGG path classification. [file 12870_2019_2046_MOESM19_ESM.tiff]

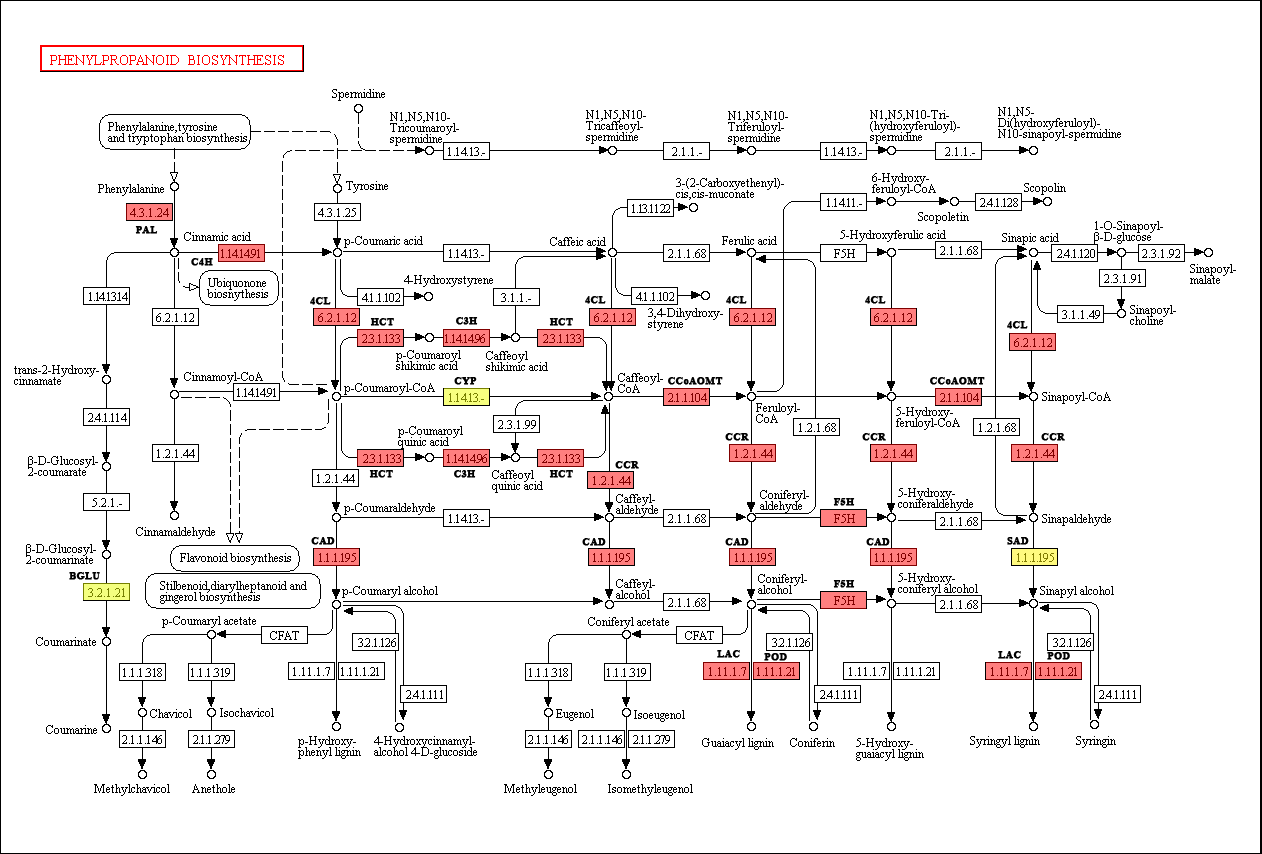

Supplement: Supplementary file 20 — Additional file 20: Figure S12. DEGs related to phenylpropanoid metabolism in the 7 DAP vs. 15 DAP comparison. Red indicates upregulation of expression, while yellow indicates no change in expression. [file 12870_2019_2046_MOESM20_ESM.tiff]

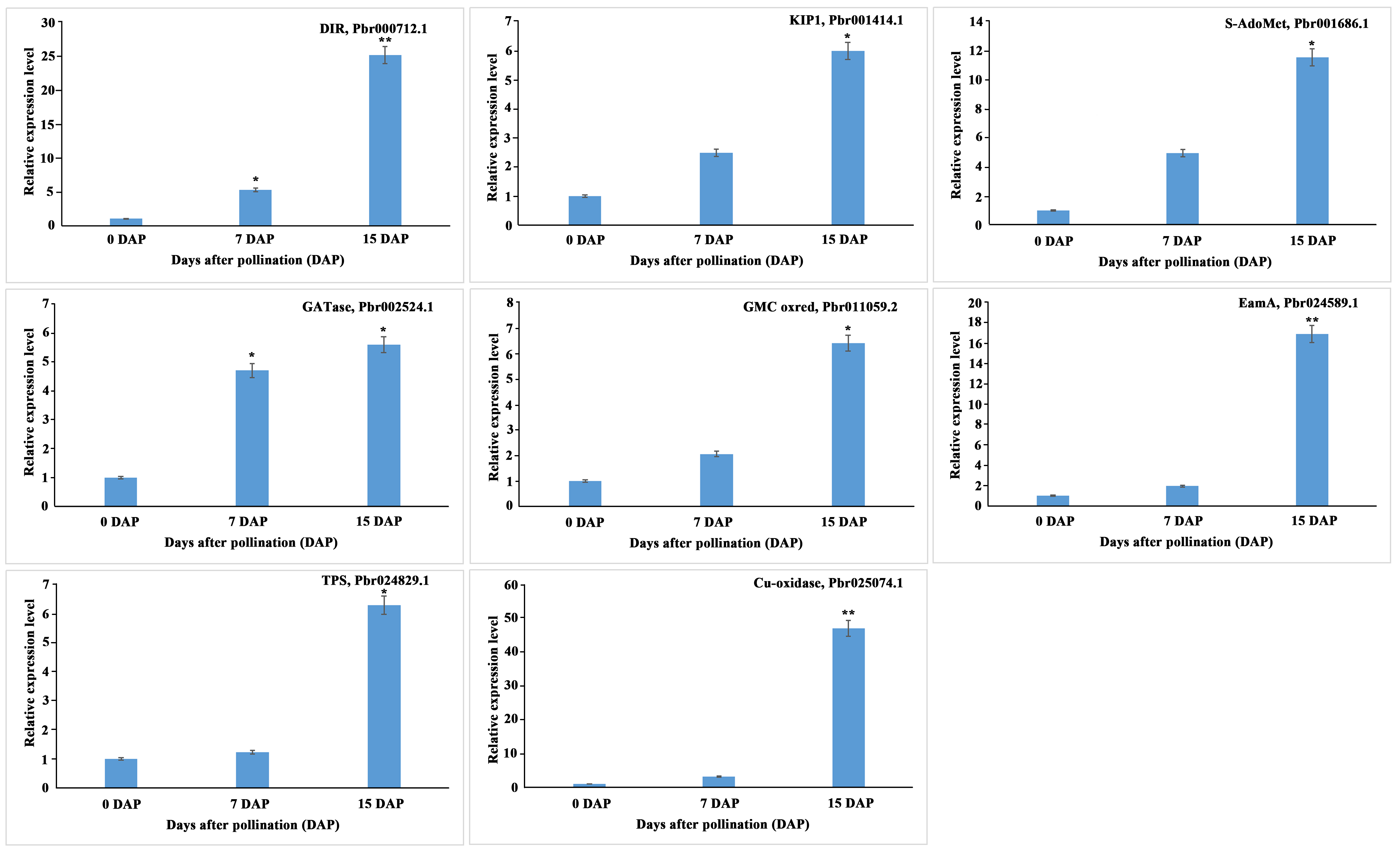

Supplement: Supplementary file 21 — Additional file 21: Figure S13. qRT-PCR validation of the expression levels of putative novel genes related to stone cell development. [file 12870_2019_2046_MOESM21_ESM.tiff]

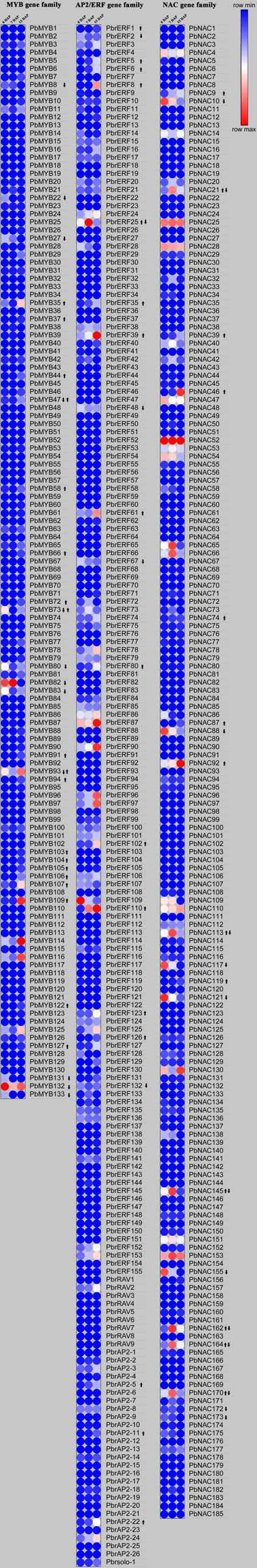

Supplement: Supplementary file 22 — Additional file 22: Figure S14. Expression profiles of the MYB, NAC, and AP2/ERF genes in Chinese white pear fruit at 0, 7, and 15 DAP. The fragments per kilobase of exon per million mapped reads (TPM) values were obtained by RNA sequencing analysis and are presented as a heat map. The colour scale shows different levels of gene expression. Red indicates a high level of expression, white signifies a medium level of expression, and blue denotes a low level of expression. [file 12870_2019_2046_MOESM22_ESM.tiff]

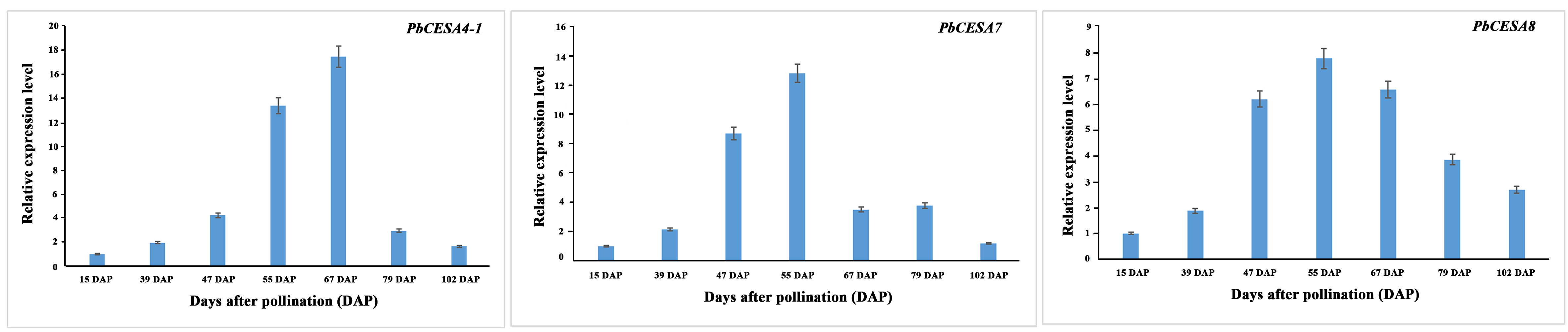

Supplement: Supplementary file 23 — Additional file 23: Figure S15. Expression patterns of PbCESA4-1, 7, 8 in fruit at different developmental stages. [file 12870_2019_2046_MOESM23_ESM.tiff]

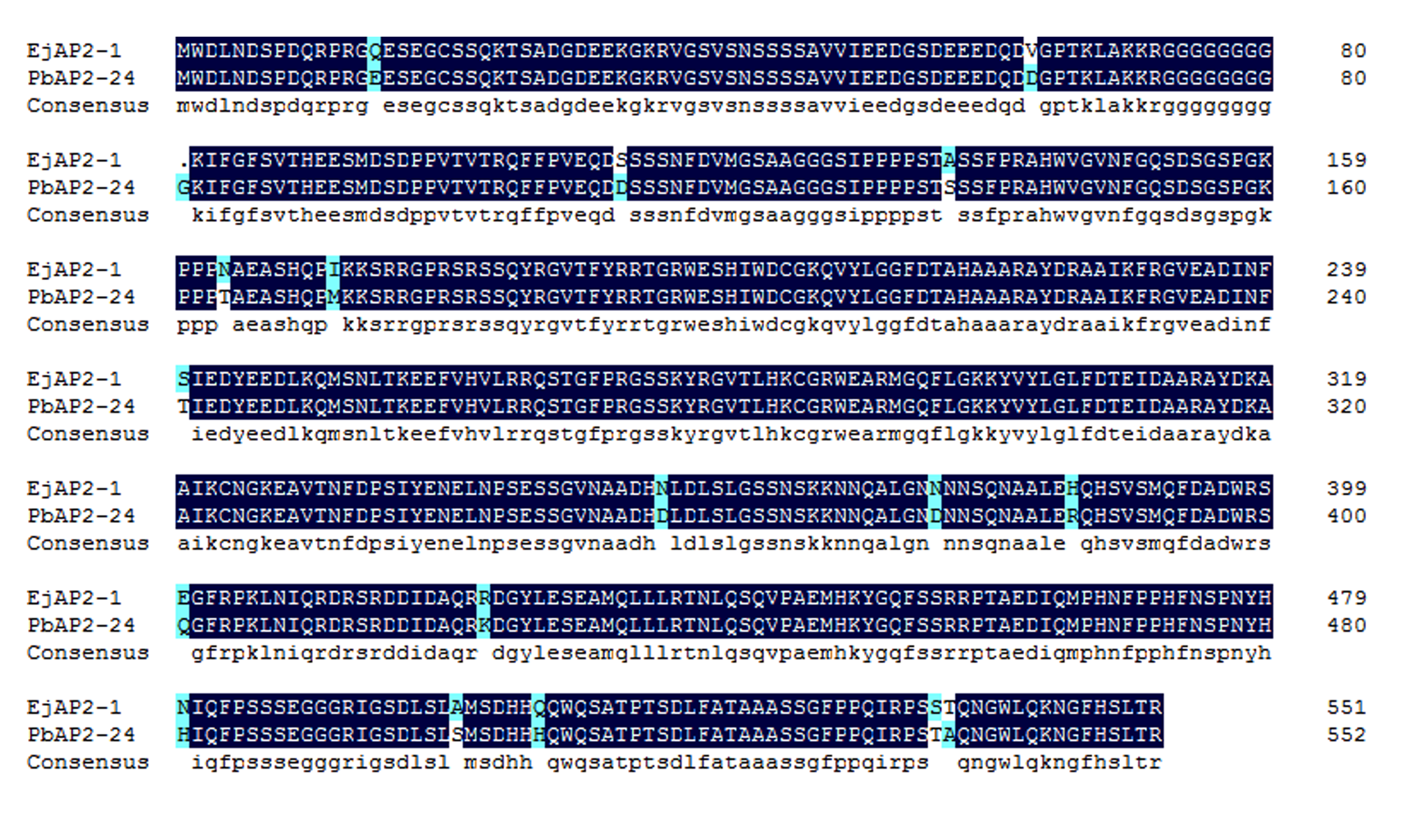

Supplement: Supplementary file 24 — Additional file 24: Figure S16. PbAP2-24 alignment with EjAP2-1 proteins. [file 12870_2019_2046_MOESM24_ESM.tiff]

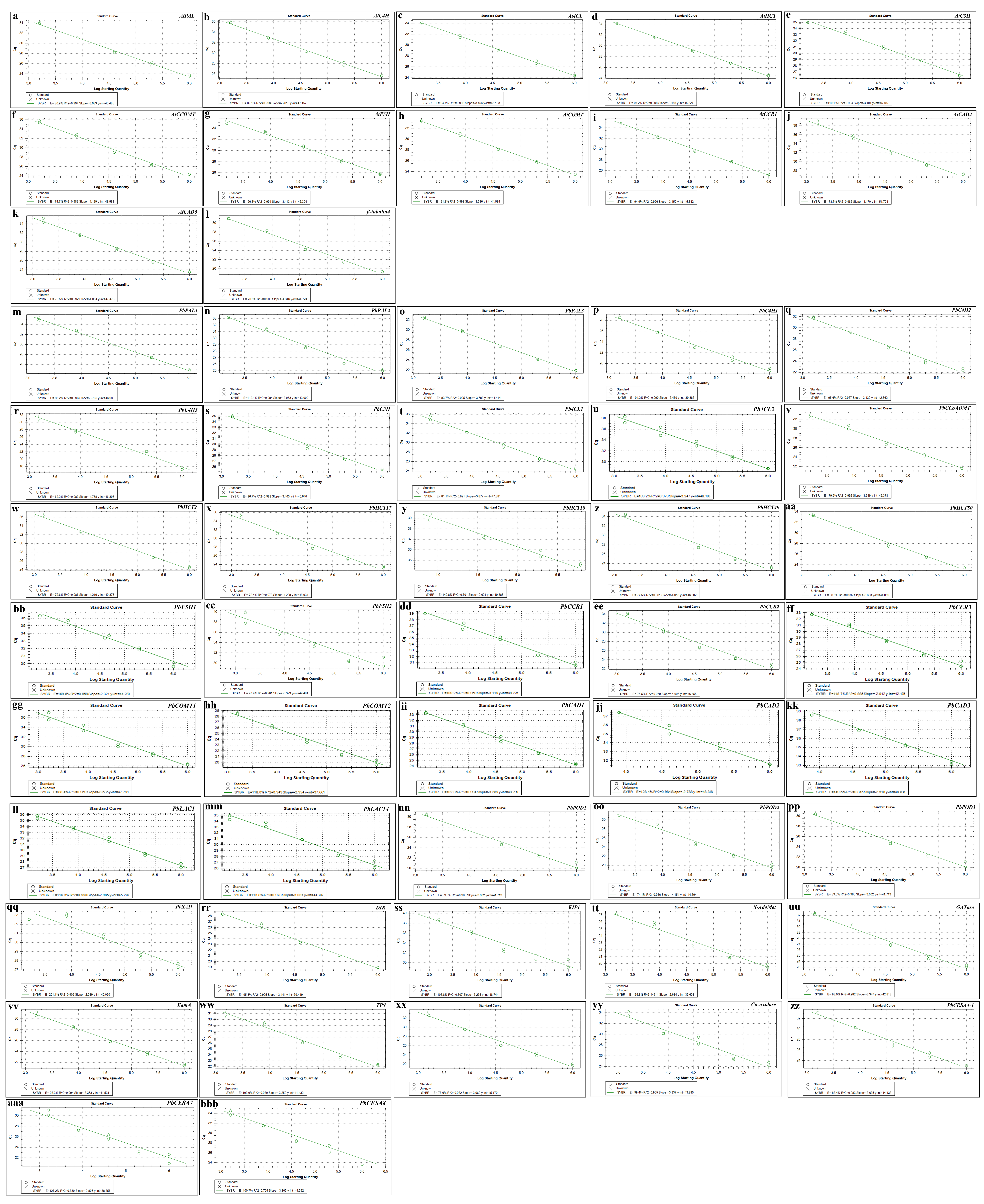

Supplement: Supplementary file 25 — Additional file 25: Figure S17. Standard curve of qRT-PCR primers. [file 12870_2019_2046_MOESM25_ESM.tiff]
